# Clinical efficacy and safety of interleukin-6 receptor antagonists (tocilizumab and sarilumab) in patients with COVID-19: a systematic review and meta-analysis

**DOI:** 10.1080/22221751.2022.2059405

**Published:** 2022-04-18

**Authors:** Su-Yeon Yu, Dae-Hyup Koh, Miyoung Choi, Seungeun Ryoo, Kyungmin Huh, Joon Sup Yeom, Young Kyung Yoon

**Affiliations:** a Division for Healthcare Technology Assessment Research, National Evidence-based Healthcare Collaborating Agency, Seoul, Republic of Korea; b Division of Infectious Diseases, Department of Medicine, Samsung Medical Center, Sungkyunkwan University School of Medicine, Seoul, Republic of Korea; c Department of Internal Medicine, Severance Hospital, Yonsei University College of Medicine, Seoul, Republic of Korea; d Division of Infectious Diseases, Department of Internal Medicine, Korea University College of Medicine, Seoul, Republic of Korea

**Keywords:** COVID-19, tocilizumab, sarilumab, meta-analysis, systematic review

## Abstract

This study investigated the efficacy and safety of interleukin-6 (IL-6) receptor antagonists with standard care treatment in patients with coronavirus disease 2019 (COVID-19). The randomized controlled trials were identified through systematic searches of electronic databases through February 10, 2022. In total, 17 trials comprising 8,614 patients were included. Compared with exclusive standard care or placebo, IL-6 receptor antagonists with standard of care treatment were associated with a significantly reduced all-cause mortality at 28 days (pooled risk ratios [RR], 0.88; 95% confidence interval (CI), 0.82–0.95; 17 studies) and progression to invasive mechanical ventilation (RR, 0.79; 95% CI, 0.71–0.88; nine studies). Particularly, the subgroup of patients with moderate-to-severe COVID-19 showed a significant mortality benefit (RR, 0.89; 95% CI, 0.81–0.96; four studies) and a reduced risk for mechanical ventilation (RR, 0.80; 95% CI, 0.70–0.91; three studies) with tocilizumab treatment. The frequency of serious adverse events was lower in the tocilizumab treatment group than in the standard of care treatment group (RR, 0.83; 95% CI, 0.71–0.97; 11 studies), with no significant difference in the sarilumab treatment group (RR, 1.12; 95% CI, 0.89–1.40; four studies). Our meta-analysis demonstrated that tocilizumab treatment showed promising results in reducing 28-day mortality and progression to mechanical ventilation in patients with moderate-to-severe COVID-19, without the burden of serious adverse events.

**Trial registration:** PROSPERO: registration number CRD42021294120.

## Introduction

The ongoing pandemic of coronavirus disease 2019 (COVID-19) has posed a major challenge for public health worldwide. Novel antiviral agents and vaccines against severe acute respiratory syndrome, which causes COVID-19, have been introduced. However, the pandemic has led to a series of epidemic waves with rapid growth in the number of infected cases, where the latter wave peaks are seen to be higher than the initial peak [1].

Clinical presentation of COVID-19 shows a notably diverse spectrum ranging from asymptomatic to acute respiratory distress syndrome (ARDS) and death. Approximately 20% of COVID-19 cases progress to moderate-to-severe disease. These cases require hospitalization and oxygen support, and 5% of cases require admission to an intensive care unit (ICU) [2].

Previous studies suggested an association between elevated pro-inflammatory cytokines and severe to critically ill patients with COVID-19 [3,4]. The early local innate immune response is the first line of antiviral defense against SARS-CoV-2 invasion. The later phase response results in dysregulated and excessive systemic immune responses, which contributes to host tissues damage and eventual mortality [5]. Particularly, the overproduction of interleukin-6 (IL-6) appears to play a prominent role in worsening the disease severity and increasing mortality in patients with COVID-19 [6].

Concurrent with the pathophysiology of COVID-19, the collaboration of virus elimination and cytokine blocking according to the illness phases, are important checkpoints to improve the therapeutic potential in patients diagnosed with COVID-19 [7]. Therefore, much interest exists in controlling the overproduction of IL-6 caused by aberrant immune activation in some patients with severe COVID-19 [8]. The National Institutes of Health (NIH) COVID-19 treatment guidelines and the expert panels of the Infectious Disease Society of America (IDSA) have suggested that administration of tocilizumab may help improve clinical outcomes in patients requiring high-flow supplemental oxygen or mechanical ventilation [9,10].

However, it is difficult to specify which population would benefit the most from tocilizumab treatment, as well as what the optimal timing and ideal dosing of administration in patients with COVID-19 would be. Furthermore, novel variants of concern are emerging continuously, and new research evidence is being updated. Safety data of immune modulation in patients with severe COVID-19 are still inadequate and needed by medical staff.

Therefore, the purpose of our study was to evaluate the efficacy and adverse events of tocilizumab and sarilumab in the treatment of COVID-19 using peer-reviewed articles, while evaluating the overall quality of synthetic evidence from a systematic review using the grading of recommendations assessment, development, and evaluation (GRADE) methodology.

## Methods

The systematic review of randomized clinical trials (RCTs) was conducted with a meta-analysis in accordance with the recommendations by the Cochrane Handbook and the preferred reporting items for systematic review and meta-analysis (PRISMA) statement [11]. The protocol for this review was prospectively registered in the International Prospective Register of Systematic Reviews (PROSPERO) under the registration number CRD42021294120.

### Search strategy

We systematically searched PubMed, Ovid-EMBASE, and CENTRAL, as well as the Korean databases (KMBASE) through Jun 14, 2021. Ongoing trials and pre-published articles were excluded. A hand search through reference lists of relevant primary and review articles were also performed for completeness. Since new evidence on the treatment for COVID-19 is continuously produced, the search was updated on the 10th day of each month starting from August 2021 to February 10, 2022. We systematically searched Ovid-MEDLINE for the search updates. The complete electronic search strategy for each database is presented in the Supplementary Material 2.

### Eligibility criteria and study selection

Articles that matched the following requirements were considered: (1) patients were adults with COVID-19; (2) interventions using IL-6 receptor antagonists (tocilizumab, sarilumab, and satralizumab); (3) the comparator was placebo or standard of care treatment; (4) outcomes including 28-day mortality, progression to invasive mechanical ventilation (IMV), and (5) the study was designed as a randomized controlled trial (RCT). Only English and Korean studies were included. Two review authors (SY and YY) independently and in duplicate evaluated publications for inclusion based on title and abstract, and then reviewed relevant full-text articles. Disagreements during the review process were addressed by consensus with the involvement of a third review author (KH).

### Risk of bias assessment and data extraction

Two writers (SY and DK) independently assessed the quality of the selected studies using the Cochrane risk of bias tool [12]. Disagreements were addressed by consensus with the participation of a third review author (MC).

Two review authors (DK and SR) extracted information from each included trial. These evaluations were carried out independently and yielded separate assessments. The disagreement was resolved by discussion and third opinion (MC). The following information was included on the data extraction form: first author, publication date, study design, characteristics of study subjects, therapeutic type of IL-6 receptor antagonist, and outcomes.

To align the included research as a single figurative criterion, some data were collected from supplementary material or, when possible, using the intention-to-treat (ITT) principle (if not defined in the original article). To obtain additional information, we also contacted the corresponding authors of the included trials regarding insufficient information.

### Rating certainty of evidence

Certainty of evidence was graded using the grading of recommendations, assessment, development, and evaluations (GRADE) approach for the primary outcomes and serious adverse events [13]. The primary outcomes included mortality at 28 days and progression to IMV. Serious adverse events, secondary infections, treatment-emergent adverse events (TEAE), time to clinical improvement, hospital discharge, time to hospital discharge, and admission to ICU were classified as secondary outcomes.

### Data synthesis and statistical analysis

For each included trial, continuous outcomes were presented as mean differences or hazard ratio (HR) with inverse-variance random-effects analysis and dichotomous outcomes as risk ratio (RR) with Mantel-Haenszel random-effects analysis and 95% confidence intervals (CIs) for all outcome measures. Because the study subjects included in each study had different demographic and clinical characteristics, the random effects model was used for all analyses to generate conservative effect estimates. Heterogeneity among trials was explored by inspecting forest plots and calculating *I*^2^ statistics.

We conducted the following pre-planned subgroup analyses: (1) IL-6 receptor antagonists therapeutics (tocilizumab and sarilumab); (2) severity of patients (moderate, severe, mild to severe, moderate to severe) stratified by the National Institute of Allergy and Infectious Diseases (NIAID) Ordinal Scale of COVID-19 Severity; mild; hospitalized, not requiring supplemental oxygen and no longer requiring ongoing medical care, moderate; hospitalized, not requiring supplemental oxygen but requiring ongoing medical care, severe; hospitalized, requiring any supplemental oxygen; requiring noninvasive ventilation or use of high-flow oxygen devices; receiving invasive mechanical ventilation or extracorporeal membrane oxygenation (ECMO) [14]. Statistical analyses were performed using Review Manager Software version 5.4. Specific stratification available in Figure S1.

Publication bias for primary endpoint was assessed through visual inspection of funnel plot. For data with an asymmetric funnel plot, Egger's linear regression test was additionally performed (Stata version 14).

## Results

### Description of included studies

**Figure 1.**
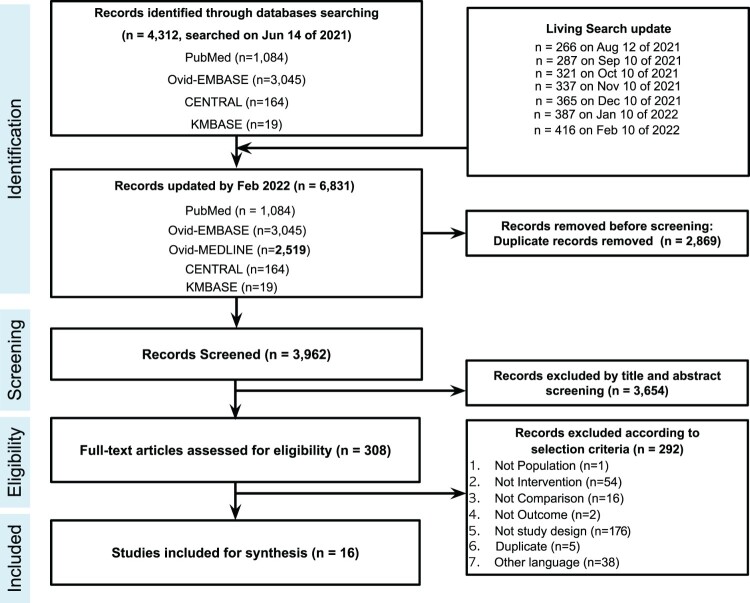
Preferred reporting items for systematic reviews and meta-analyses (PRISMA) study flowchart.

A total of 6,831 articles were retrieved from the databases, resulting in 3,962 articles after excluding duplicates. According to the selection criteria, 308 articles were selected for full-text review. 16 published articles with 17 RCTs (Hermine 2022 reported two separate RCTs) were finally included in this systematic review (8,614 patients) [15–30]. Details of the study selections and the flowchart of the review are shown in Figure 1. Of the 17 final selected studies, ten studies used tocilizumab treatment, five studies used sarilumab treatment, and one study compared tocilizumab or sarilumab treatment with the control. One study used tocilizumab treatment as the combination group with remdesivir. Regarding patient severity, the studies found were as follows: eight severe, two moderate, five moderate-to-severe, and one mild-to-severe. Specific characteristics of studies included are presented in Table 1. The results of the risk of bias summary are shown in Supplementary Material 3. Most studies showed low risks of bias. GRADE evidence profiles and a summary of findings are described in Table 2.

**Table 1.** Baseline study characteristics of published randomized controlled trials of IL-6 receptor antagonist [15–30].

First author	Trial name Trial number	Treatment dose (mg)^a^	Control (mg)	steroid usage (%)^e^	Patients at randomization (N)	Age, (y)	Respiratory status*	Patient severity	Published date^d^
Declercq [15]	COV-AID NCT04330638	TCZ (8, max800)	SOC	TCZ (62) Control (63)	229	TCZ (65) Control (64)	PaO2/FiO2 155 (92-247) (TCZ) 111 (88-250) (Control)	MODERATE-TO-SEVERE	Lancet Respir Med Dec 2021
Gordon [16]	REMAP-CAP NCT02735707	TCZ (8^a^, max800) SARI (400)	SOC	TCZ (92.7) SARI (95.8) Control (93.9)	895	TCZ (61.5) SARI (63.4) Control (61.1)	PaO2/FiO2 115 (89-162) (TCZ) 126 (99-157) (SARI) 118 (89-169) (Control)	SEVERE	N. Eng. J. Med. Apr 2021
Hermine [17]	CORIMUNO-TOCI-1 NCT04331808	TCZ (8^a^)	SOC	TCZ (46) Control (73)	131	TCZ (64) Control (63.3)	SpO2 95 (93–96) (TCZ) 95 (93–97) (Control)	SEVERE	JAMA Intern. Med. Jan 2021
Hermine [18]	CORIMUNO-TOCI-2 NCT04331808	TCZ (8, max800)	SOC	TCZ (41) Control (40)	97	TCZ (63.2) Control (65.4)	PaO2/FiO2 128 (100-175) (TCZ) 138 (191-187) (Control)	SEVERE	Eur. Respir. J Feb 2022
	CORIMUNO-SARI-2 NCT04324073	SARI (200)	SOC	SARI (21) Control (21)	91	SARI (61.9) Control (61.2)	PaO2/FiO2 132 (106-204) (SARI) 102 (86-155) (Control)		
Horby [19]	RECOVERY	TCZ (8^a^) 400–800^b^	SOC	TCZ (82) Control (82)	4116	TCZ (63.3) Control (63.9)	SpO2 94 (92–96) (TCZ) 94 (91–95) (Control)	MODERATE-TO-SEVERE	Lancet May 2021
Lescure [21]	REGENERON-P3 NCT04327388	SARI 200 SARI 400	Placebo	SARI 200 (36.5) SARI 400 (45.1) Placebo (46.4)	420	SARI 200 (58) SARI 400 (58) Placebo (60)	SpO2 95 (93–96) (SARI 200) 94 (93–96) (SARI 400) 94 (93–96) (Control)	SEVERE	Lancet Respir. Med. May 2021
Mariette [20]	CORIMUNO-SARI-1 NCT04324073	SARI 400	SOC	SARI (15) Control (25)	148	SARI (61.7) Control (62.8)	SpO2 94 (92–96) (SARI) 94 (93–96) (Control)	SEVERE	The Lancet Rheumatology Nov 2021
Merchante 2022 [22]	SARICOR NCT04357860	SARI 200 SARI 400	SOC	SARI 200 (89) SARI 400 (92) Placebo (88)	118	SARI 200 (65) SARI 400 (57) Placebo (57)	SpO2 92 (89–96) (SARI 200) 92 (89–95) (SARI 400) 93 (90–95) (Control)	MODERATE-TO-SEVERE	Antimicrob. Agents Chemother Feb 2022
Rosas [23]	COVACTA NCT04320615	TCZ (8^a^, max 800),	Placebo	TCZ (33.7) Placebo (52.1)	452	TCZ (60.9) Placebo (60.6)	N/A^c^	MODERATE-TO-SEVERE	N. Eng. J. Med. Apr 2021
Rosas [24]	REMDACTA NCT04409262	TCZ (8^a^) + RDV (100 daily)	Placebo + RDV (100 daily)	TCZ + RDV (88.1) Placebo + RDV (88.3)	649	TCZ + RDV (60.1) Placebo + RDV (58.2)	N/A^c^	SEVERE	Intensive Care Med. OCT 2021
Salama [25]	EMPACTA NCT04372186	TCZ (8^a^, max 800)	Placebo	TCZ (80.3) Placebo (87.5)	389	TCZ (56) Placebo (55.6)	N/A^c^	MODERATE	N. Eng. J. Med. Jan 2021
Salvarani [26]	RCT-TCZ-COVID-19 NCT04346355	TCZ (8^a^, max 800)	SOC	TCZ (10.0) Control (11.1)	126	TCZ (61.5) Control (60.0)	PaO2/FIO2 262.5 (241.0-286.5) (TCZ) 268.2 (244.0-290.0) (Control)	MODERATE	JAMA Intern. Med. Oct 2020
Sancho-López [27]	SARTRE EudraCT 2020-002037-15	SARI 200-400^b^	SOC	SARI (100) Control (100)	201	SARI (60) Control (60)	N/A^c^	SEVERE	Infect Dis Ther Dec 2021
Soin [28]	COVINTOC CTRI/2020/05/025369	TCZ (6^a^, max 480)	SOC	TCZ (91) Control (91)	180	TCZ (56) Control (54)	N/A^c^	MODERATE-TO-SEVERE	Lancet. Respir. Med. Mar 2021
Stone [29]	BACC-Bay NCT04356937	TCZ (8^a^, max 800)	Placebo	TCZ (11) Placebo (6)	243	TCZ (61.6) Placebo (56.5)	N/A^c^	MILD-TO-SEVERE	N. Eng. J. Med. Oct 2020
Veiga [30]	TOCIBRAS NCT04403685	TCZ (8^a^, max 800)	SOC	TCZ (83.6) Control (88.7)	129	TCZ (57.4) Control (57.5)	SpO2 95 (92–96) (TCZ) 95 (93–96) (Control)	SEVERE	BMJ Jan 2021

Abbreviations: TCZ, tocilizumab; SARI, sarilumab; RDV, remdesivir; SpO_2_, Oxygen saturation; PaO_2_/FiO_2_, Partial pressure of arterial oxygen/fraction of inspired oxygen; PRISMA, Preferred Reporting Items for Systematic Reviews and Meta-Analysis; SOC, Standard of Care including antibiotic agents, antiviral agents, corticosteroids, vasopressor support, anticoagulants.

^a^ mg/kg.

^b^ depending on weight.

^c^ Not Available, not reported as patient characteristics at baseline.

^d^ standard abbreviations, ISO 4.

^e^ includes systemic corticosteroid or Glucocorticoids *Oxygen saturation (SpO_2_, median (IQR), %), PaO_2_/FiO_2_ (P/F ratio, median (IQR), mmHg).

**Table 2.** GRADE summary of findings table of mortality, progression to invasive mechanical ventilation, and serious adverse events.

Outcomes	Subgroups	Anticipated absolute effects* (95% CI)		Relative effect (95% CI)	№ of participants (studies)	Certainty of the evidence (GRADE)
		Risk with standard care/placebo	Risk with IL-6 receptor antagonists			
Mortality at 28 days	Total	307 per 1,000	270 per 1,000 (252–291)	RR 0.88 (0.82–0.95)	8455 (17 studies)	⨁⨁⨁⨁ High
	Tocilizumab	290 per 1,000	258 per 1,000 (238–276)	RR 0.89 (0.82–0.95)	7369 (12 studies)	⨁⨁⨁⨁ High
	Sarilumab	227 per 1,000	184 per 1,000 (118–263)	RR 0.81 (0.59–1.10)	1483 (6 studies)	⨁⨁⨁◯ Moderate^a^
Progression to IMV	Total	210 per 1,000	166 per 1,000 (149–187)	RR 0.79 (0.71–0.89)	5507 (9 studies)	⨁⨁⨁⨁ High
	Tocilizumab	211 per 1,000	167 per 1,000 (150–186)	RR 0.79 (0.71–0.88)	5392 (8 studies)	⨁⨁⨁⨁ High
	Sarilumab	103 per 1,000	118 per 1,000 (39–360)	RR 1.15 (0.38–3.51)	115 (1 studies)	⨁⨁⨁◯ Moderate^a^
Serious adverse events	Total	234 per 1,000	209 per 1,000 (183–239)	RR 0.89 (0.78–1.02)	3952 (15 studies)	⨁⨁⨁◯ Moderate^b^
	Tocilizumab	210 per 1,000	175 per 1,000 (149–204)	RR 0.83 (0.71–0.97)	3263 (11 studies)	⨁⨁⨁⨁ High
	Sarilumab	131 per 1,000	147 per 1,000 (117–184)	RR 1.12 (0.89–1.40)	1091 (4 studies)	⨁⨁⨁◯ Moderate^a^

Abbreviation: IL-6: interleukin-6; CI: Confidence interval; RR: Risk ratio; IMV: invasive mechanical ventilation.

GRADE Working Group grades of evidence.

**High certainty**: we are very confident that the true effect lies close to that of the estimate of the effect.

**Moderate certainty**: we are moderately confident in the effect estimate: the true effect is likely to be close to the estimate of the effect, but there is a possibility that it is substantially different.

The risk in the intervention group (and its 95% CI) is based on the assumed risk in the comparison group and the relative effect of the intervention (and its 95% CI)

^a^ Imprecision downgraded by 1 level due to low number of events and a wide confidence interval consistent with the possibility for benefit and the possibility for harm

^b^ Imprecision downgraded by 1 level due to a wide confidence interval consistent with the possibility for benefit and the possibility for harm.

### Primary outcomes

#### 28-day Mortality

Seventeen clinical trials reported 28-day mortality. IL-6 receptor antagonists were significantly associated with reduced all-cause 28-day mortality among 8,455 patients (duplicated control group patients in REMAP-CAP study excluded) compared with the control group, RR 0.88 (95% CI 0.82–0.95; *I*^2^ 0%; high certainty evidence). While tocilizumab significantly reduced all-cause 28-day mortality, RR 0.89 (95% CI 0.82–0.95; *I*^2^ 0%; 12 studies; high certainty evidence), the effect of sarilumab is uncertain, RR 0.81 (95% CI 0.59–1.10; *I*^2^ 0%; six studies; moderate certainty evidence) as well as described in Table 2 and Figure S2.

**Figure 2.**
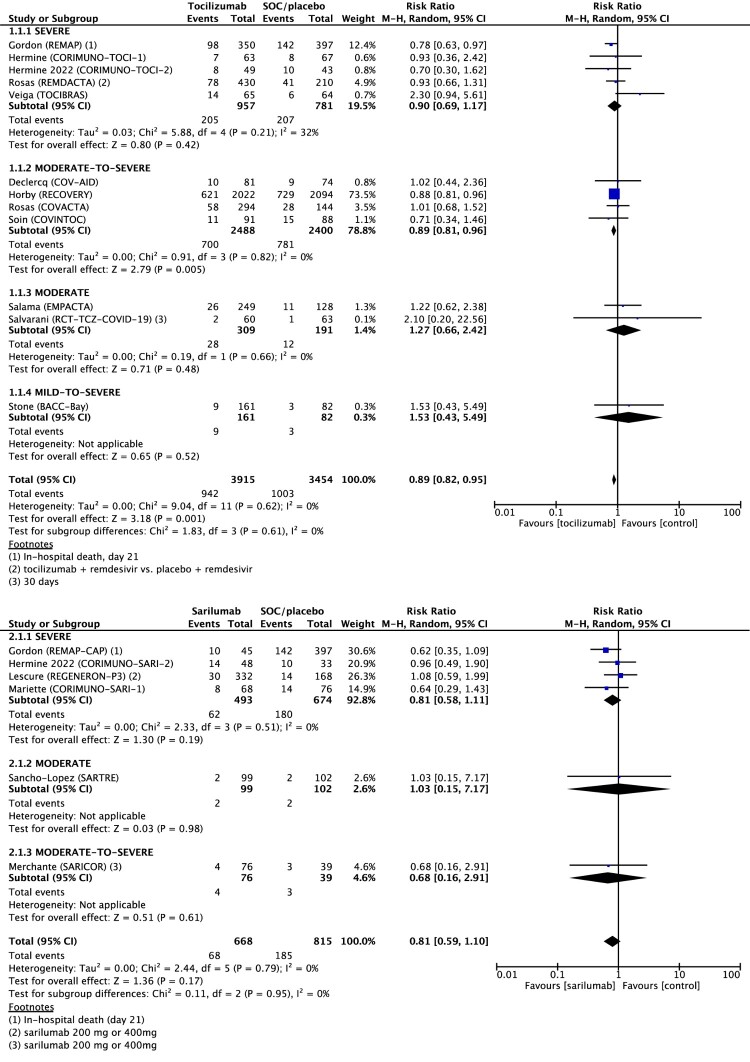
Forest plot of all-cause 28-day mortality. Forest plot showing the risk ratio in mortality between patients treated with IL-6 receptor antagonist compared with standard of care (SOC). Meta-analysis on 17 randomized controlled studies comprising 8455 patients showed that mortality was significantly 11% lower for patients with COVID-19 treated with tocilizumab compared to SOC and not significant but with lowering 19% mortality in patients treated with sarilumab. Abbreviations: CI, confidence interval; M-H, Mantel-Haenszel random-effects; SOC, standard of care.

Sub-group analysis with patients’ severity was followed. Tocilizumab showed different effects on all-cause 28-day mortality according to the severity aligned with the moderate-to-severe group treated with tocilizumab. This group had significantly lower mortality than the control group: severe, RR 0.90 (95% CI 0.69–1.17, *I*^2^ 32%, five studies); moderate-to-severe, RR 0.89 (95% CI 0.81–0.96, *I*^2^ 0%, four studies); moderate, RR 1.27 (95% CI 0.66–2.42; *I*^2^ 0%; two studies); mild-to-severe, RR 1.53 (95% CI 0.43–5.49, one study). Sarilumab had no significant effect on all-cause 28-day mortality in any severity group: severe, RR 0.81 (95% CI 0.58–1.14, *I*^2^ 0%, four studies); moderate, RR 1.03 (95% CI 0.15–7.17, one study); moderate-to-severe, RR 0.68 (95% CI 0.16–2.91, one study) (Figure 2).

The publication bias of the included studies was judged at low risk in the domain of primary endpoint. Although the funnel plot is asymmetric, Egger’s test did not show statistically significant publication bias (*P* = 0.21) (Supplementary Material 4).

#### Progression to IMV

The progression to IMV was reported in nine clinical trials. IL-6 receptor antagonists were significantly associated with reduced progression to IMV among patients compared with the control group, RR 0.79 (95% CI 0.71–0.89; *I*^2^ 0%; high certainty evidence). While tocilizumab significantly reduced all-cause 28-day mortality, RR 0.79 (95% CI 0.71–0.88; *I*^2^ 0%; 8 studies; high certainty evidence), the effect of sarilumab is uncertain, RR 1.15 (95% CI 0.38–3.51; one study; moderate certainty evidence) (Figure S3).

**Figure 3.**
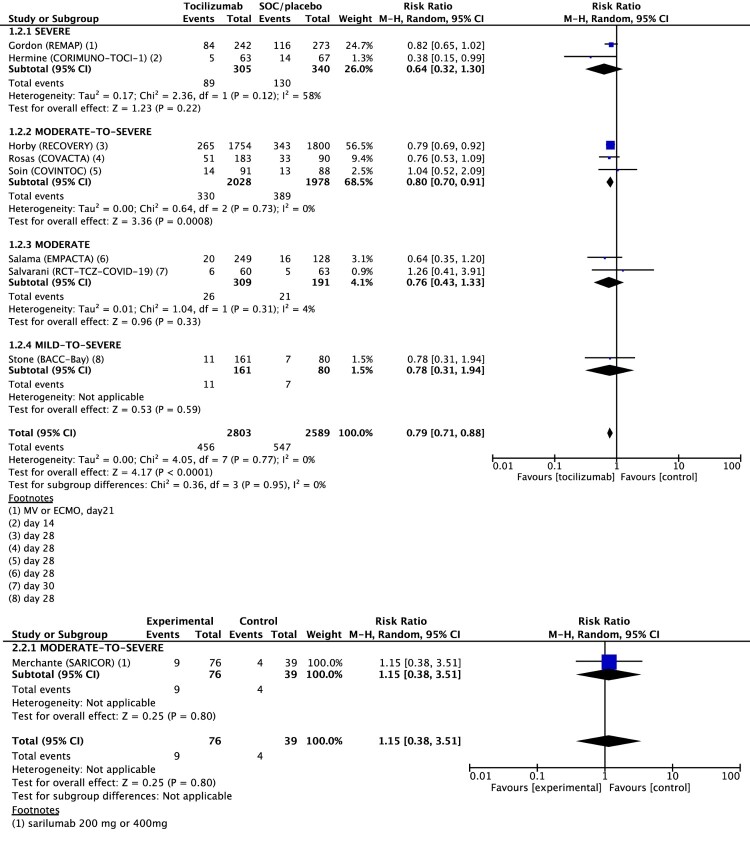
Forest plot of progression to invasive mechanical ventilation. Forest plot showing the risk ratio in progression to invasive mechanical ventilation (IMV) between patients treated with tocilizumab compared with standard of care (SOC). Meta-analysis on 9 randomized controlled studies comprising 5507 patients showed that progression to IMV was significantly 21% lower for patients with COVID-19 treated with tocilizumab compared to SOC, and not significantly different in patients treated with sarilumab. Abbreviations: CI, confidence interval; M-H, Mantel-Haenszel random-effects; SOC, standard of care; IMV, invasive mechanical ventilation.

Sub-group analysis with patients’ severity was followed with progression to IMV and moderate-to-severe group treated with tocilizumab had significantly lower Progression to IMV than control group, which is consistent with mortality analysis: RR 0.80 (95% CI, 0.70–0.91). (Figure 3)

### Secondary outcomes

The secondary outcomes were serious adverse events, secondary infections, TEAE, the cases and time to hospital discharge, the cases of admission to ICU, or time to clinical improvement.

**Figure 4.**
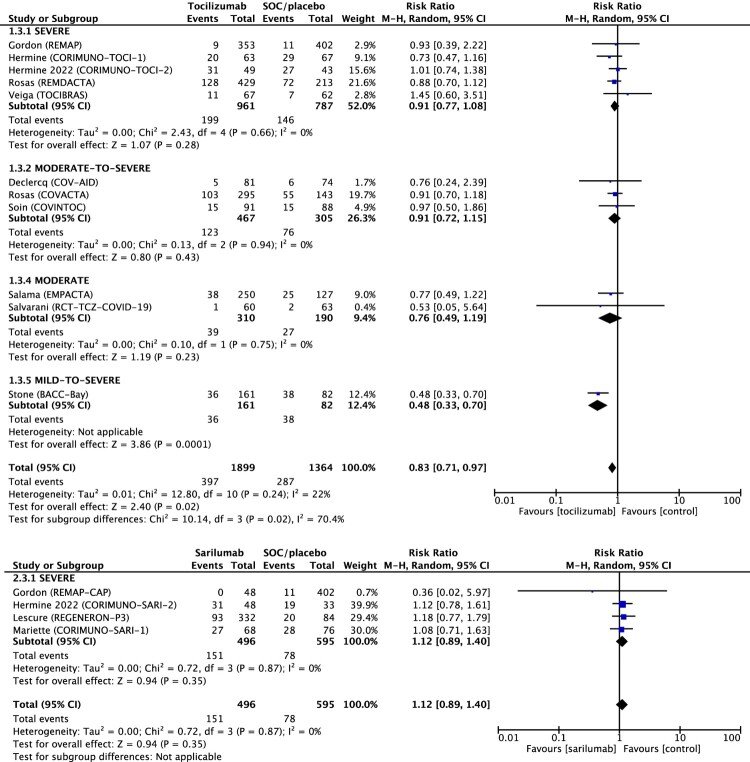
Forest plot of serious adverse events. Forest plot showing the risk ratio in serious adverse events between patients treated with IL-6 receptor antagonist compared with standard of care (SOC). Meta-analysis on 15 randomized controlled studies comprising 3952 patients showed that serious adverse events was significantly 17% lower for patients with COVID-19 treated with tocilizumab compared to SOC and not significant in patients treated with sarilumab. Abbreviations: CI, confidence interval; M-H, Mantel-Haenszel random-effects; SOC, standard of care.

IL-6 receptor antagonists were not significantly associated with reducing serious adverse events among 3,952 patients: RR 0.89 (95% CI 0.78–1.02) (Figure S4), while tocilizumab treatment significantly reduced serious adverse events with RR 0.83 (95% CI 0.71–0.97) (Figure 4). Specifically, cases of secondary infection were not significant in the sub-group analysis with RR 0.82 (95% CI 0.66–1.01) as well as TEAE with RR 1.02 (95% CI 0.95–1.08) (Figure S5 and Figure S6). Time to discharge was reduced with the Hazard Ratio of 1.29 (95% CI 1.12–1.48) and cases of Hospital discharge; RR 1.08 (95% CI 1.01–1.15) in favour of the intervention group (Figure S7 and Figure S8).

In the cases of admission to ICU and time to clinical improvement, both the tocilizumab group and the sarilumab group also did not show any significant effect on those outcomes in the sub-group analysis (Figure S9 and Figure S10).

## Discussion

Consistent with our findings, recent meta-analyses of randomized trials exploring the effect of tocilizumab treatment in patients with COVID-19 showed a favourable effect on mortality risk [31–35]. Some meta-analyses suggested that tocilizumab treatment had no significant positive effect on mortality, but these studies included non-randomized trials [36,37]. However, not all patients with COVID-19 will consistently have a favourable effect on clinical outcome from the treatment of IL-6 receptor antagonists. This is likely due to the heterogeneous nature of disease severity, concomitant medication, the levels of inflammatory markers; and dosage, timing, and duration of tocilizumab or sarilumab treatment.

Our study found a significant benefit of tocilizumab treatment with patients with moderate-to-severe COVID-19 in 28-day mortality and progression to IMV. In our analysis, the maximum clinical benefit of mortality from tocilizumab was seen in the COVID-19 patients with the NIAID ordinal scale of COVID-19 severity ≥5, which was similar to the findings of previous studies [16,17,19,28]. Compared with previous studies that showed contradictory results, our study differed with respect to the target study population. Those studies included a relatively small number of patients requiring minimal high-flow oxygen supplementation or those who did not require oxygen therapy [23,25,29,30].

A previous meta-analysis showed that a favourable effect of IL-6 receptor antagonists on treatment outcome appeared to be more marked in patients receiving corticosteroids at randomization compared to those who did not [35]. In accordance with the results, the REMAP-CAP trial and RECOVERY trial in which more than 80% of the study subjects concomitantly treated with corticosteroids, revealed that improved survival rates in patients treated with tocilizumab [16,19]. Furthermore, in one study (excluding patients with mechanical ventilation or admission to the ICU), tocilizumab treatment contributed to the improvement in mortality rate, although the number of patients receiving corticosteroids was as low as 46% [17]. However, other studies that included all such patients showed no improvement in mortality with tocilizumab treatment when only 11–69% of patients received glucocorticoids [23,29,30]. Although the role of tocilizumab in combination with remdesivir is unclear [24], corticosteroids were already found to be associated with a reduction in mortality in patients with severe COVID-19 [38]. Because of the lack of solid beneficial evidence for the routine use of corticosteroids in general COVID-19 patients, more evidence on its potential role as a single or combined treatment in management of COVID-19 should be available [39].

Unlike the NIH COVID-19 guidelines that uniformly recommend tocilizumab treatment for patients admitted to the ICU, the IDSA guidelines recommend tocilizumab only for patients requiring supplemental oxygen with elevated inflammatory markers [9,10]. However, the majority of RCTs did not select patients to receive tocilizumab based on elevated inflammatory markers [16,17,23,25,30]. On the other hand, in the 3 studies, tocilizumab was administered based on the elevation of C-reactive protein [19,29,30]. Tocilizumab was associated with a lower mortality rate in studies that included patients with CRP ≥75 mg/L [19], but not in studies that included patients with CRP ≥50 mg/L [29,30]. In the former study, the concomitant administration rate of corticosteroids was 82%, whereas the latter was only 11% and 69%, respectively [19,29,30].

The REMAP-CAP trial and RECOVERY trials have suggested that a beneficial effect of tocilizumab treatment was associated with early administration in the clinical course of illness: 2 days of hospitalization in RECOVERY and <24 h in the ICU for REMAP-CAP [16,19]. A previous study supported a clinical benefit of early tocilizumab treatment, within the first day of intubation, and a possible detriment to later administration [40]. Studies suggest that tocilizumab improves treatment outcomes in enrolled patients about 10 days after symptoms onset and within 48 h from hospital admission, with a moderate to severe illness [16,17,19]. A recent meta-analysis also suggested that administration of tocilizumab within 10 days after symptom onset could reduce mortality and the need for intubation [33].

In our study, the use of sarilumab treatment did not improve the clinical outcomes, unlike tocilizumab treatment. However, the relatively small number of trials related to sarilumab treatment limited the evaluation of sarilumab treatment in an optimized subset of COVID-19 patients. Despite these findings, the sarilumab therapy was not associated with significant serious adverse events. Furthermore, some studies have shown that the possibility of benefit from the administration of sarilumab treatment in targeted patient groups cannot be ruled out [19,21]. Therefore, the subsequent randomized clinical trials, as well as meta-analyses, are needed to warrant determining the usefulness of sarilumab treatment for improving treatment outcomes of COVID-19 patients.

No safety concerns associated with IL-6 receptor antagonists were observed in our analysis. Contrary to usual expectations, secondary infections were not increased with administration of Il-6 receptor antagonists. Serious adverse events even occurred in fewer patients in the tocilizumab treatment group in combination with the standard of care, compared with exclusive standard of care alone or placebo group. These findings are presumed to be the result of shortened hospital stay and reduced exposure to invasive procedures such as IMV through tocilizumab administration. Furthermore, there was no significant difference in serious adverse events according to the use of sarilumab.

Our study had several limitations. First, dosage, timing, and duration of IL-6 receptor antagonists had heterogeneity. However, most included studies comprehended that the patients received the standard dose: tocilizumab, 8 mg per kilogram of body weight (one or two doses) and sarilumab, 200 mg for patients <75 kg body weight or 400 mg for patients ≥75 kg body weight (single dose). Furthermore, our study included only RCTs that were published in peer-reviewed journals to strengthen the validity of the studies. Second, our study did not explore several confounding variables that could affect treatment outcomes other than IL-6 receptor antagonists. However, the heterogeneity of the study subjects was minimized by stratification of clinical severity. Third, balanced use of comedication or intervention after randomization was not guaranteed in this analysis.

In conclusion, this study suggests a potential therapeutic role of tocilizumab treatment in patients with moderate-to-severe COVID-19, during the early period since the onset of clinical deterioration, notably when concomitantly administered with corticosteroid therapy. Given the current dynamic changes at the molecular level of SARS-CoV-2, the meta-analysis of IL-6 receptor antagonists should be constantly updated.

## Supplementary Material

Supplemental Material
