# Supplementary material for: Clinical efficacy and safety of interleukin-6 receptor antagonists (tocilizumab and sarilumab) in patients with COVID-19: a systematic review and meta-analysis
Source: Emerg Microbes Infect. 2022 Apr 18;11(1):1154–65. doi: 10.1080/22221751.2022.2059405 (PMC9037226; doi:10.1080/22221751.2022.2059405)
Supplement: Supplemental Material [file TEMI_A_2059405_SM8986.zip › Suppl files/SM 2 Search_strategy.docx]

PubMed

| **no.** | **Search strategy** |
| --- | --- |
| #1 | "COVID-19"[Mesh] |
| #2 | "COVID-19"[TW] OR "COVID 19"[TW] OR "COVID-19 Virus Disease"[TW] OR "COVID 19 Virus Disease"[TW] OR "COVID-19 Virus Diseases"[TW] OR "Disease, COVID-19 Virus"[TW] OR "Virus Disease, COVID-19"[TW] OR "COVID-19 Virus Infection"[TW] OR "COVID 19 Virus Infection"[TW] OR "COVID-19 Virus Infections"[TW] OR "Infection, COVID-19 Virus"[TW] OR "Virus Infection, COVID-19"[TW] OR "2019-nCoV Infection"[TW] OR "2019 nCoV Infection"[TW] OR "2019-nCoV Infections"[TW] OR "Infection, 2019-nCoV"[TW] OR "Coronavirus Disease-19"[TW] OR "Coronavirus Disease 19"[TW] OR "2019-nCoV Disease"[TW] OR "2019 nCoV Disease"[TW] OR "2019-nCoV Diseases"[TW] OR "Disease, 2019-nCoV"[TW] OR "COVID19"[TW] OR "Coronavirus Disease 2019"[TW] OR "Disease 2019, Coronavirus"[TW] OR "SARS Coronavirus 2 Infection"[TW] OR "SARS-CoV-2 Infection"[TW] OR "Infection, SARS-CoV-2"[TW] OR "SARS CoV 2 Infection"[TW] OR "SARS-CoV-2 Infections"[TW] OR "COVID-19 Pandemic"[TW] OR "COVID 19 Pandemic"[TW] OR "COVID-19 Pandemics"[TW] OR "Pandemic, COVID-19"[TW] OR "2019 Novel Coronavirus Disease"[TW] OR "2019 Novel Coronavirus Infection"[TW] OR "coronavirus disease 2"[TW] OR "coronavirus disease 2019 pneumonia"[TW] OR "coronavirus infection 2019"[TW] OR "COVID"[TW] OR "COVID 19 induced pneumonia"[TW] OR "COVID 2019"[TW] OR "COVID-19 induced pneumonia"[TW] OR "COVID-19 pneumonia"[TW] OR "nCoV 2019 disease"[TW] OR "nCoV 2019 infection"[TW] OR "paucisymptomatic coronavirus disease 2019"[TW] OR "SARS coronavirus 2 pneumonia"[TW] OR "SARSCoV2 disease"[TW] OR "SARS-CoV2 disease"[TW] OR "SARS-CoV-2 disease"[TW] OR "SARSCoV2 infection"[TW] OR "SARS-CoV2 infection"[TW] OR "SARS-CoV-2 pneumonia"[TW] OR "severe acute respiratory syndrome 2"[TW] OR "severe acute respiratory syndrome 2 pneumonia"[TW] OR "severe acute respiratory syndrome coronavirus 2 infection"[TW] OR "severe acute respiratory syndrome coronavirus 2019 infection"[TW] OR "severe acute respiratory syndrome CoV-2 infection"[TW] OR "Wuhan coronavirus disease"[TW] OR "Wuhan coronavirus infection"[TW] OR "2019 novel coronavirus epidemic"[TW] OR "new coronavirus pneumonia"[TW] OR "novel coronavirus 2019 disease"[TW] OR "novel coronavirus 2019 infection"[TW] OR "novel coronavirus disease 2019"[TW] OR "novel coronavirus infected pneumonia"[TW] OR "novel coronavirus infection 2019"[TW] OR "novel coronavirus pneumonia"[TW] OR "2019nCoV"[TW] OR "19nCoV"[TW] OR "COVID19*"[TW] OR "SARSCOV-2"[TW] OR "SARSCOV2"[TW] OR "corona virus 2"[TW] OR "Wuhan"[TW] OR "Hubei"[TW] OR "new coronavirus"[TW] OR "novel coronavirus"[TW] OR "novel corona virus"[TW] OR "novel CoV"[TW] |
| #3 | "SARS-CoV-2"[Mesh] |
| #4 | "SARS-CoV-2"[TW] OR "Coronavirus Disease 2019 Virus"[TW] OR "Wuhan Seafood Market Pneumonia Virus"[TW] OR "SARS-CoV-2 Virus"[TW] OR "SARS CoV 2 Virus"[TW] OR "SARS-CoV-2 Viruses"[TW] OR "Virus, SARS-CoV-2"[TW] OR "2019-nCoV"[TW] OR "COVID-19 Virus"[TW] OR "COVID 19 Virus"[TW] OR "COVID-19 Viruses"[TW] OR "Virus, COVID-19"[TW] OR "Wuhan Coronavirus"[TW] OR "Coronavirus, Wuhan"[TW] OR "SARS Coronavirus 2"[TW] OR "Coronavirus 2, SARS"[TW] OR "Severe Acute Respiratory Syndrome Coronavirus 2"[TW] OR "2019 Novel Coronavirus"[TW] OR "2019 Novel Coronaviruses"[TW] OR "Coronavirus, 2019 Novel"[TW] OR "Novel Coronavirus, 2019"[TW] OR "2019 nCOV"[TW] OR "2019 severe acute respiratory syndrome coronavirus 2"[TW] OR "HCoV-19"[TW] OR "Human coronavirus 2019"[TW] OR "nCoV-2019"[TW] OR "SARS2 (virus)"[TW] OR "SARS-related coronavirus 2"[TW] OR "Sever acute respiratory syndrome coronavirus 2"[TW] OR "Severe acute respiratory coronavirus 2"[TW] OR "Severe acute respiratory syndorme coronavirus 2"[TW] OR "severe acute respiratory syndrome 2 virus"[TW] OR "severe acute respiratory syndrome corona virus 2"[TW] OR "severe acute respiratory syndrome coronavirus 2019"[TW] OR "Severe acute respiratory syndrome coronoavirus 2"[TW] OR "Severe acute respiratory syndrome coronvirus 2"[TW] OR "severe acute respiratory syndrome CoV-2 virus"[TW] OR "Severe acute respiratory syndrome related coronavirus 2"[TW] OR "Severe acute respiratory syndrome virus 2"[TW] OR "Severe acute respiratoy syndrome coronavirus 2"[TW] OR "2019 new coronavirus"[TW] OR "novel 2019 coronavirus"[TW] OR "novel coronavirus-19"[TW] |
| **#5** | **#1 OR #2 OR #3 OR #4** |
| #6 | "tocilizumab"[Supplementary Concept] |
| #7 | "Tocilizumab"[TW] OR "RHPM-1"[TW] OR "RG-1569"[TW] OR "R-1569"[TW] OR "MSB11456"[TW] OR "MSB-11456"[TW] OR "atlizumab"[TW] OR "monoclonal antibody, MRA"[TW] OR "RO-4877533"[TW] OR "Actemra"[TW] OR "Roactemra"[TW] OR "actemra 200"[TW] OR "lusinex"[TW] OR "r 1569"[TW] OR "r1569"[TW] |
| #8 | "sarilumab"[Supplementary Concept] |
| #9 | "sarilumab"[TW] OR "SAR-153191"[TW] OR "SAR153191"[TW] OR "Kevzara"[TW] OR "REGN-88"[TW] OR "REGN88"[TW] OR "kevzara sc"[TW] OR "regn 88"[TW] OR "sar 153191"[TW] |
| #10 | "satralizumab"[Supplementary Concept] |
| #11 | "satralizumab"[TW] OR "enspryng"[TW] OR "rg 6168"[TW] OR "rg6168"[TW] OR "ro 5333787"[TW] OR "ro5333787"[TW] OR "sa 237"[TW] OR "sa237"[TW] OR "sapelizumab"[TW] |
| #12 | "interleukin-6 inhibitor"[TW] OR "IL-6 inhibitor"[TW] OR "interleukin-6 receptor antagonist"[TW] OR "IL-6 receptor antagonist"[TW] |
| **#13** | **#6 OR #7 OR #8 OR #9 OR #10 OR #11 OR #12** |
| **#14** | **#5 AND #13** |
| **#15** | **#14 NOT ("animals"[MeSH] NOT "Humans"[MeSH])** |
| **#16** | **#15 AND (2020/6/1:2021/12/31[pdat])** |

Ovid-EMBASE

| **no.** | **Search strategy** |
| --- | --- |
| #1 | exp coronavirus disease 2019/ |
| #2 | ("COVID-19" OR "COVID 19" OR "COVID-19 Virus Disease" OR "COVID 19 Virus Disease" OR "COVID-19 Virus Diseases" OR "Disease, COVID-19 Virus" OR "Virus Disease, COVID-19" OR "COVID-19 Virus Infection" OR "COVID 19 Virus Infection" OR "COVID-19 Virus Infections" OR "Infection, COVID-19 Virus" OR "Virus Infection, COVID-19" OR "2019-nCoV Infection" OR "2019 nCoV Infection" OR "2019-nCoV Infections" OR "Infection, 2019-nCoV" OR "Coronavirus Disease-19" OR "Coronavirus Disease 19" OR "2019-nCoV Disease" OR "2019 nCoV Disease" OR "2019-nCoV Diseases" OR "Disease, 2019-nCoV" OR "COVID19" OR "Coronavirus Disease 2019" OR "Disease 2019, Coronavirus" OR "SARS Coronavirus 2 Infection" OR "SARS-CoV-2 Infection" OR "Infection, SARS-CoV-2" OR "SARS CoV 2 Infection" OR "SARS-CoV-2 Infections" OR "COVID-19 Pandemic" OR "COVID 19 Pandemic" OR "COVID-19 Pandemics" OR "Pandemic, COVID-19" OR "2019 Novel Coronavirus Disease" OR "2019 Novel Coronavirus Infection" OR "coronavirus disease 2" OR "coronavirus disease 2019 pneumonia" OR "coronavirus infection 2019" OR "COVID" OR "COVID 19 induced pneumonia" OR "COVID 2019" OR "COVID-19 induced pneumonia" OR "COVID-19 pneumonia" OR "nCoV 2019 disease" OR "nCoV 2019 infection" OR "paucisymptomatic coronavirus disease 2019" OR "SARS coronavirus 2 pneumonia" OR "SARSCoV2 disease" OR "SARS-CoV2 disease" OR "SARS-CoV-2 disease" OR "SARSCoV2 infection" OR "SARS-CoV2 infection" OR "SARS-CoV-2 pneumonia" OR "severe acute respiratory syndrome 2" OR "severe acute respiratory syndrome 2 pneumonia" OR "severe acute respiratory syndrome coronavirus 2 infection" OR "severe acute respiratory syndrome coronavirus 2019 infection" OR "severe acute respiratory syndrome CoV-2 infection" OR "Wuhan coronavirus disease" OR "Wuhan coronavirus infection" OR "2019 novel coronavirus epidemic" OR "new coronavirus pneumonia" OR "novel coronavirus 2019 disease" OR "novel coronavirus 2019 infection" OR "novel coronavirus disease 2019" OR "novel coronavirus infected pneumonia" OR "novel coronavirus infection 2019" OR "novel coronavirus pneumonia" OR "2019nCoV" OR "19nCoV" OR "COVID19$" OR "SARSCOV-2" OR "SARSCOV2" OR "corona virus 2" OR "Wuhan" OR "Hubei" OR "new coronavirus" OR "novel coronavirus" OR "novel corona virus" OR "novel CoV").ti,ab,kw. |
| #3 | exp Severe acute respiratory syndrome coronavirus 2/ |
| #4 | ("SARS-CoV-2" OR "Coronavirus Disease 2019 Virus" OR "Wuhan Seafood Market Pneumonia Virus" OR "SARS-CoV-2 Virus" OR "SARS CoV 2 Virus" OR "SARS-CoV-2 Viruses" OR "Virus, SARS-CoV-2" OR "2019-nCoV" OR "COVID-19 Virus" OR "COVID 19 Virus" OR "COVID-19 Viruses" OR "Virus, COVID-19" OR "Wuhan Coronavirus" OR "Coronavirus, Wuhan" OR "SARS Coronavirus 2" OR "Coronavirus 2, SARS" OR "Severe Acute Respiratory Syndrome Coronavirus 2" OR "2019 Novel Coronavirus" OR "2019 Novel Coronaviruses" OR "Coronavirus, 2019 Novel" OR "Novel Coronavirus, 2019" OR "2019 nCOV" OR "2019 severe acute respiratory syndrome coronavirus 2" OR "HCoV-19" OR "Human coronavirus 2019" OR "nCoV-2019" OR "SARS2 (virus)" OR "SARS-related coronavirus 2" OR "Sever acute respiratory syndrome coronavirus 2" OR "Severe acute respiratory coronavirus 2" OR "Severe acute respiratory syndorme coronavirus 2" OR "severe acute respiratory syndrome 2 virus" OR "severe acute respiratory syndrome corona virus 2" OR "severe acute respiratory syndrome coronavirus 2019" OR "Severe acute respiratory syndrome coronoavirus 2" OR "Severe acute respiratory syndrome coronvirus 2" OR "severe acute respiratory syndrome CoV-2 virus" OR "Severe acute respiratory syndrome related coronavirus 2" OR "Severe acute respiratory syndrome virus 2" OR "Severe acute respiratoy syndrome coronavirus 2" OR "2019 new coronavirus" OR "novel 2019 coronavirus" OR "novel coronavirus-19").ti,ab,kw. |
| **#5** | **1 OR 2 OR 3 OR 4** |
| #6 | exp tocilizumab/ |
| #7 | ("Tocilizumab" OR "RHPM-1" OR "RG-1569" OR "R-1569" OR "MSB11456" OR "MSB-11456" OR "atlizumab" OR "monoclonal antibody, MRA" OR "RO-4877533" OR "Actemra" OR "Roactemra" OR "actemra 200" OR "lusinex" OR "r 1569" OR "r1569").ti,ab,kw. |
| #8 | exp sarilumab/ |
| #9 | ("sarilumab" OR "SAR-153191" OR "SAR153191" OR "Kevzara" OR "REGN-88" OR "REGN88" OR "kevzara sc" OR "regn 88" OR "sar 153191").ti,ab,kw. |
| #10 | exp satralizumab/ |
| #11 | ("satralizumab" OR "enspryng" OR "rg 6168" OR "rg6168" OR "ro 5333787" OR "ro5333787" OR "sa 237" OR "sa237" OR "sapelizumab").ti,ab,kw. |
| #12 | ("interleukin-6 inhibitor" OR "IL-6 inhibitor" OR "interleukin-6 receptor antagonist" OR "IL-6 receptor antagonist").ti,ab,kw. |
| **#13** | **6 OR 7 OR 8 OR 9 OR 10 OR 11 OR 12** |
| **#14** | **5 AND 13** |
| **#15** | **14 not ((exp animal/ or animal experiment/ or nonhuman/) not (exp human/ or human experiment/))** |
| **#16** | **limit 15 to dc=20200601-20211231** |

Cochrane

| **no.** | **Search strategy** |
| --- | --- |
| #1 | [mh "COVID-19"] |
| #2 | "COVID-19":ti,ab,kw OR "COVID 19":ti,ab,kw OR "COVID-19 Virus Disease":ti,ab,kw OR "COVID 19 Virus Disease":ti,ab,kw OR "COVID-19 Virus Diseases":ti,ab,kw OR "Disease, COVID-19 Virus":ti,ab,kw OR "Virus Disease, COVID-19":ti,ab,kw OR "COVID-19 Virus Infection":ti,ab,kw OR "COVID 19 Virus Infection":ti,ab,kw OR "COVID-19 Virus Infections":ti,ab,kw OR "Infection, COVID-19 Virus":ti,ab,kw OR "Virus Infection, COVID-19":ti,ab,kw OR "2019-nCoV Infection":ti,ab,kw OR "2019 nCoV Infection":ti,ab,kw OR "2019-nCoV Infections":ti,ab,kw OR "Infection, 2019-nCoV":ti,ab,kw OR "Coronavirus Disease-19":ti,ab,kw OR "Coronavirus Disease 19":ti,ab,kw OR "2019-nCoV Disease":ti,ab,kw OR "2019 nCoV Disease":ti,ab,kw OR "2019-nCoV Diseases":ti,ab,kw OR "Disease, 2019-nCoV":ti,ab,kw OR "COVID19":ti,ab,kw OR "Coronavirus Disease 2019":ti,ab,kw OR "Disease 2019, Coronavirus":ti,ab,kw OR "SARS Coronavirus 2 Infection":ti,ab,kw OR "SARS-CoV-2 Infection":ti,ab,kw OR "Infection, SARS-CoV-2":ti,ab,kw OR "SARS CoV 2 Infection":ti,ab,kw OR "SARS-CoV-2 Infections":ti,ab,kw OR "COVID-19 Pandemic":ti,ab,kw OR "COVID 19 Pandemic":ti,ab,kw OR "COVID-19 Pandemics":ti,ab,kw OR "Pandemic, COVID-19":ti,ab,kw OR "2019 Novel Coronavirus Disease":ti,ab,kw OR "2019 Novel Coronavirus Infection":ti,ab,kw OR "coronavirus disease 2":ti,ab,kw OR "coronavirus disease 2019 pneumonia":ti,ab,kw OR "coronavirus infection 2019":ti,ab,kw OR "COVID":ti,ab,kw OR "COVID 19 induced pneumonia":ti,ab,kw OR "COVID 2019":ti,ab,kw OR "COVID-19 induced pneumonia":ti,ab,kw OR "COVID-19 pneumonia":ti,ab,kw OR "nCoV 2019 disease":ti,ab,kw OR "nCoV 2019 infection":ti,ab,kw OR "paucisymptomatic coronavirus disease 2019":ti,ab,kw OR "SARS coronavirus 2 pneumonia":ti,ab,kw OR "SARSCoV2 disease":ti,ab,kw OR "SARS-CoV2 disease":ti,ab,kw OR "SARS-CoV-2 disease":ti,ab,kw OR "SARSCoV2 infection":ti,ab,kw OR "SARS-CoV2 infection":ti,ab,kw OR "SARS-CoV-2 pneumonia":ti,ab,kw OR "severe acute respiratory syndrome 2":ti,ab,kw OR "severe acute respiratory syndrome 2 pneumonia":ti,ab,kw OR "severe acute respiratory syndrome coronavirus 2 infection":ti,ab,kw OR "severe acute respiratory syndrome coronavirus 2019 infection":ti,ab,kw OR "severe acute respiratory syndrome CoV-2 infection":ti,ab,kw OR "Wuhan coronavirus disease":ti,ab,kw OR "Wuhan coronavirus infection":ti,ab,kw OR "2019 novel coronavirus epidemic":ti,ab,kw OR "new coronavirus pneumonia":ti,ab,kw OR "novel coronavirus 2019 disease":ti,ab,kw OR "novel coronavirus 2019 infection":ti,ab,kw OR "novel coronavirus disease 2019":ti,ab,kw OR "novel coronavirus infected pneumonia":ti,ab,kw OR "novel coronavirus infection 2019":ti,ab,kw OR "novel coronavirus pneumonia":ti,ab,kw OR "2019nCoV":ti,ab,kw OR "19nCoV":ti,ab,kw OR "COVID19*":ti,ab,kw OR "SARSCOV-2":ti,ab,kw OR "SARSCOV2":ti,ab,kw OR "corona virus 2":ti,ab,kw OR "Wuhan":ti,ab,kw OR "Hubei":ti,ab,kw OR "new coronavirus":ti,ab,kw OR "novel coronavirus":ti,ab,kw OR "novel corona virus":ti,ab,kw OR "novel CoV":ti,ab,kw |
| #3 | [mh "SARS-CoV-2"] |
| #4 | "SARS-CoV-2":ti,ab,kw OR "Coronavirus Disease 2019 Virus":ti,ab,kw OR "Wuhan Seafood Market Pneumonia Virus":ti,ab,kw OR "SARS-CoV-2 Virus":ti,ab,kw OR "SARS CoV 2 Virus":ti,ab,kw OR "SARS-CoV-2 Viruses":ti,ab,kw OR "Virus, SARS-CoV-2":ti,ab,kw OR "2019-nCoV":ti,ab,kw OR "COVID-19 Virus":ti,ab,kw OR "COVID 19 Virus":ti,ab,kw OR "COVID-19 Viruses":ti,ab,kw OR "Virus, COVID-19":ti,ab,kw OR "Wuhan Coronavirus":ti,ab,kw OR "Coronavirus, Wuhan":ti,ab,kw OR "SARS Coronavirus 2":ti,ab,kw OR "Coronavirus 2, SARS":ti,ab,kw OR "Severe Acute Respiratory Syndrome Coronavirus 2":ti,ab,kw OR "2019 Novel Coronavirus":ti,ab,kw OR "2019 Novel Coronaviruses":ti,ab,kw OR "Coronavirus, 2019 Novel":ti,ab,kw OR "Novel Coronavirus, 2019":ti,ab,kw OR "2019 nCOV":ti,ab,kw OR "2019 severe acute respiratory syndrome coronavirus 2":ti,ab,kw OR "HCoV-19":ti,ab,kw OR "Human coronavirus 2019":ti,ab,kw OR "nCoV-2019":ti,ab,kw OR "SARS2 (virus)":ti,ab,kw OR "SARS-related coronavirus 2":ti,ab,kw OR "Sever acute respiratory syndrome coronavirus 2":ti,ab,kw OR "Severe acute respiratory coronavirus 2":ti,ab,kw OR "Severe acute respiratory syndorme coronavirus 2":ti,ab,kw OR "severe acute respiratory syndrome 2 virus":ti,ab,kw OR "severe acute respiratory syndrome corona virus 2":ti,ab,kw OR "severe acute respiratory syndrome coronavirus 2019":ti,ab,kw OR "Severe acute respiratory syndrome coronoavirus 2":ti,ab,kw OR "Severe acute respiratory syndrome coronvirus 2":ti,ab,kw OR "severe acute respiratory syndrome CoV-2 virus":ti,ab,kw OR "Severe acute respiratory syndrome related coronavirus 2":ti,ab,kw OR "Severe acute respiratory syndrome virus 2":ti,ab,kw OR "Severe acute respiratoy syndrome coronavirus 2":ti,ab,kw OR "2019 new coronavirus":ti,ab,kw OR "novel 2019 coronavirus":ti,ab,kw OR "novel coronavirus-19":ti,ab,kw |
| **#5** | **#1 OR #2 OR #3 OR #4** |
| #6 | "Tocilizumab":ti,ab,kw OR "RHPM-1":ti,ab,kw OR "RG-1569":ti,ab,kw OR "R-1569":ti,ab,kw OR "MSB11456":ti,ab,kw OR "MSB-11456":ti,ab,kw OR "atlizumab":ti,ab,kw OR "monoclonal antibody, MRA":ti,ab,kw OR "RO-4877533":ti,ab,kw OR "Actemra":ti,ab,kw OR "Roactemra":ti,ab,kw OR "actemra 200":ti,ab,kw OR "lusinex":ti,ab,kw OR "r 1569":ti,ab,kw OR "r1569":ti,ab,kw |
| #7 | "sarilumab":ti,ab,kw OR "SAR-153191":ti,ab,kw OR "SAR153191":ti,ab,kw OR "Kevzara":ti,ab,kw OR "REGN-88":ti,ab,kw OR "REGN88":ti,ab,kw OR "kevzara sc":ti,ab,kw OR "regn 88":ti,ab,kw OR "sar 153191":ti,ab,kw |
| #8 | "satralizumab":ti,ab,kw OR "enspryng":ti,ab,kw OR "rg 6168":ti,ab,kw OR "rg6168":ti,ab,kw OR "ro 5333787":ti,ab,kw OR "ro5333787":ti,ab,kw OR "sa 237":ti,ab,kw OR "sa237":ti,ab,kw OR "sapelizumab":ti,ab,kw |
| #9 | "interleukin-6 inhibitor":ti,ab,kw OR "IL-6 inhibitor":ti,ab,kw OR "interleukin-6 receptor antagonist":ti,ab,kw OR "IL-6 receptor antagonist":ti,ab,kw |
| **#10** | **#6 OR #7 OR #8 OR #9** |
| **#11** | **#5 AND #10** |
| **#12** | **#11 with Cochrane Library publication date from Jun 2020 to Dec 2021** |

KMBASE

| **no.** | **Search strategy** |
| --- | --- |
| #1 | ([ALL=COVID-19] OR [ALL=COVID19] OR [ALL=coronavirus] OR [ALL=SARS-CoV-2] OR [ALL=Severe acute respiratory syndrome coronavirus 2]) AND ([ALL=Tocilizumab] OR [ALL=Sarilumab] OR [ALL=Satralizumab]) |
| #2 | ([ALL=코로나-19] OR [ALL=코로나19] OR [ALL=코로나]) AND ([ALL=Tocilizumab] OR [ALL=Sarilumab] OR [ALL=Satralizumab]) |
| **#3** | **#1 OR #2** |
| **#4** | **The number of duplicates removed from Endnote. / Duplication: 1** |
