# Supplementary material for: Clinical efficacy and safety of interleukin-6 receptor antagonists (tocilizumab and sarilumab) in patients with COVID-19: a systematic review and meta-analysis
Source: Emerg Microbes Infect. 2022 Apr 18;11(1):1154–65. doi: 10.1080/22221751.2022.2059405 (PMC9037226; doi:10.1080/22221751.2022.2059405)

Supplementary Figure 1. patient severity stratification

| **Severity** | **NIAID 8-point**  **Ordinal scale** | | **Hospitalized** | **Oxygen Therapy/**  **medical care** | **IMV** | **MV or ECMO** |
| --- | --- | --- | --- | --- | --- | --- |
|  | **Score** | **Definition** |  |  |  |  |
| **1. Uninfected** | **1** | **not hospitalized and no limitations of activities** | **X** |  |  |  |
| **2. Ambulatory**  **(Mild)** | **2** | **not hospitalized, with limitation of activities, home oxygen requirement, or both** | **X** |  |  |  |
|  |  |  |  |  |  |  |
|  | **3** | **hospitalized, not requiring supplemental oxygen, and no longer requiring ongoing medical care** | **O** | **X/X** |  |  |
| **3. Moderate** | **4** | **hospitalized, not requiring supplemental oxygen but requiring ongoing medical care** | **O** | **X/O** |  |  |
|  |  |  |  |  |  |  |
| **4. Severe** | **5** | **hospitalized, requiring any supplemental oxygen** | **O** | **O/O** |  |  |
|  |  |  |  |  |  |  |
|  | **6** | **hospitalized, requiring noninvasive ventilation or use of high-flow oxygen devices** | **O** |  | **O** |  |
|  |  |  |  |  |  |  |
|  |  |  |  |  |  |  |
|  | **7** | **hospitalized, receiving invasive mechanical ventilation (IMV) or extracorporeal membrane oxygenation (ECMO)** | **O** |  |  | **O** |
| **5. Critical** |  |  |  |  |  |  |
|  | **8** | **Death** |  |  |  |  |

Supplementary Figure 2. mortality at day 28


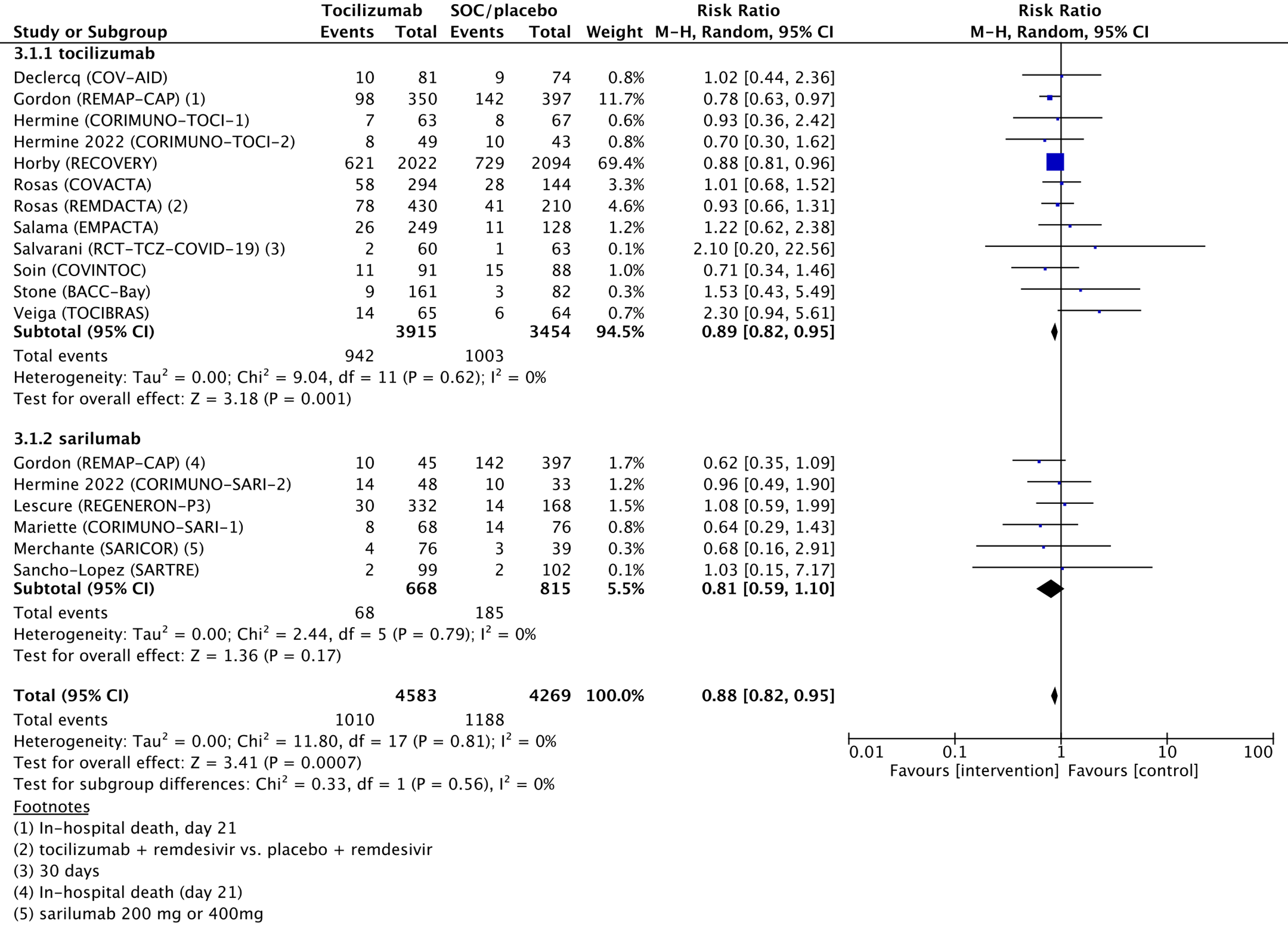


Supplementary Figure 3. progression to invasive mechanical ventilation


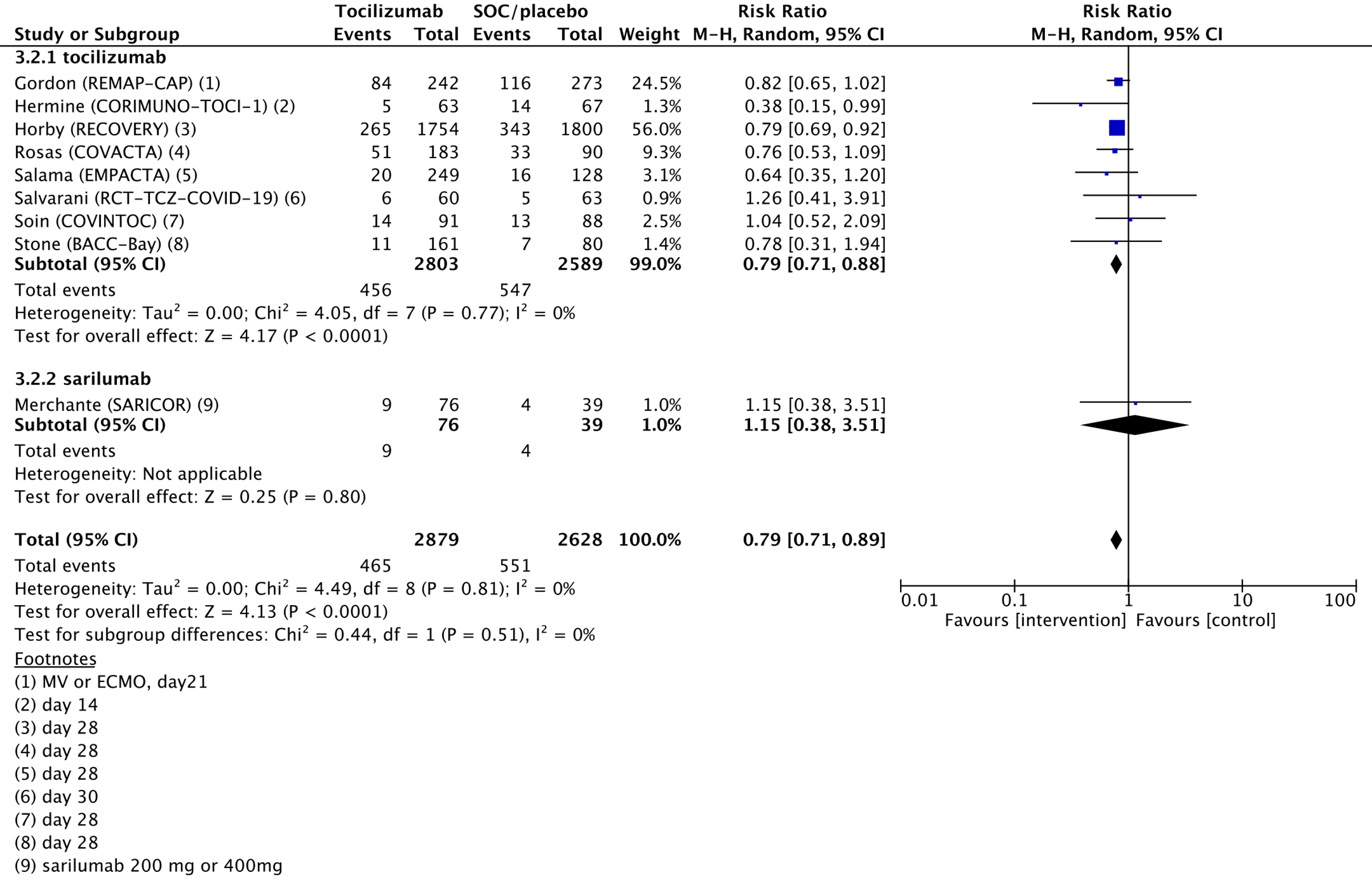


Supplementary Figure 4. serious adverse events


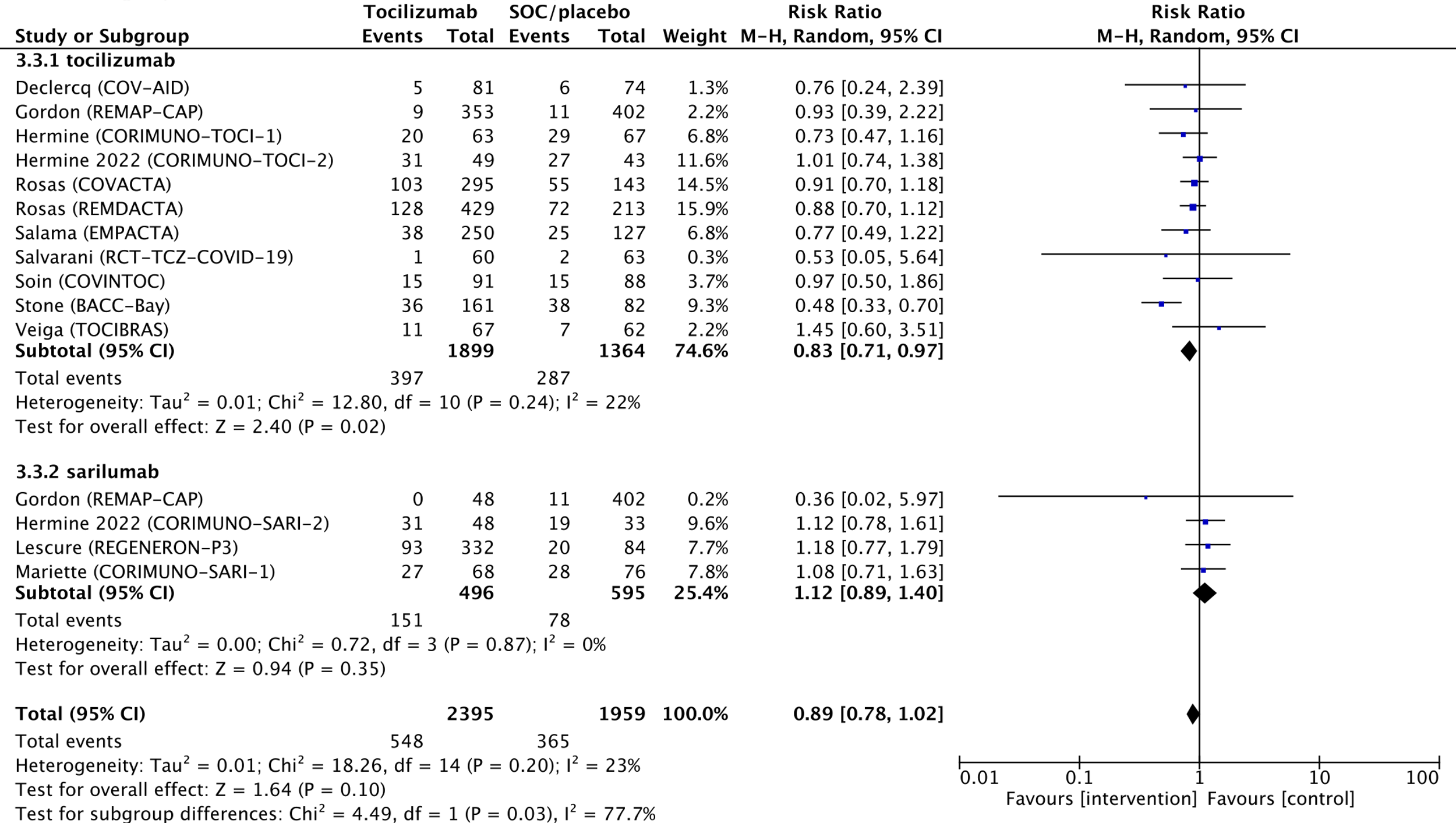


Supplementary Figure 5. secondary infections


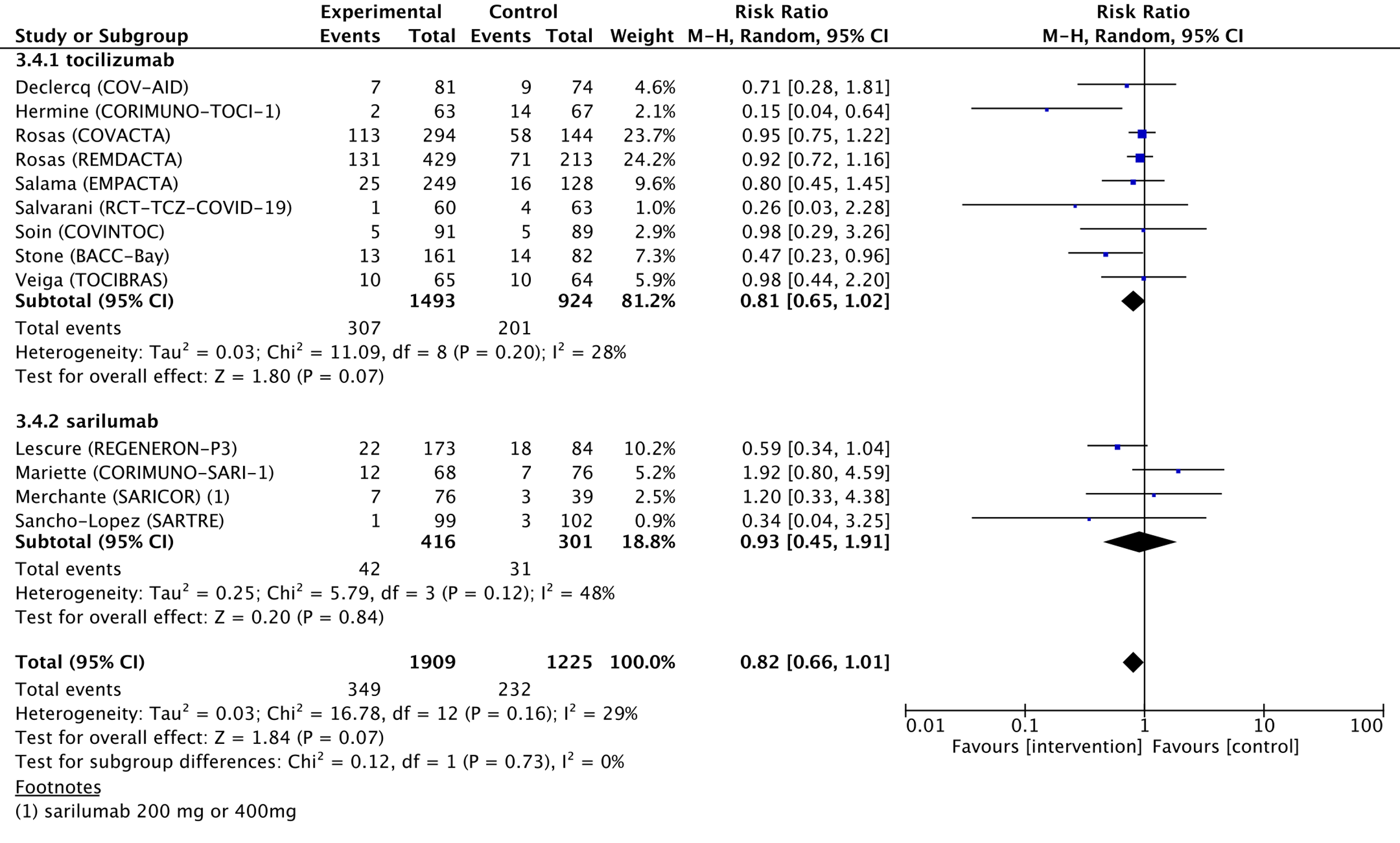


Supplementary Figure 6. treatment emergent adverse event


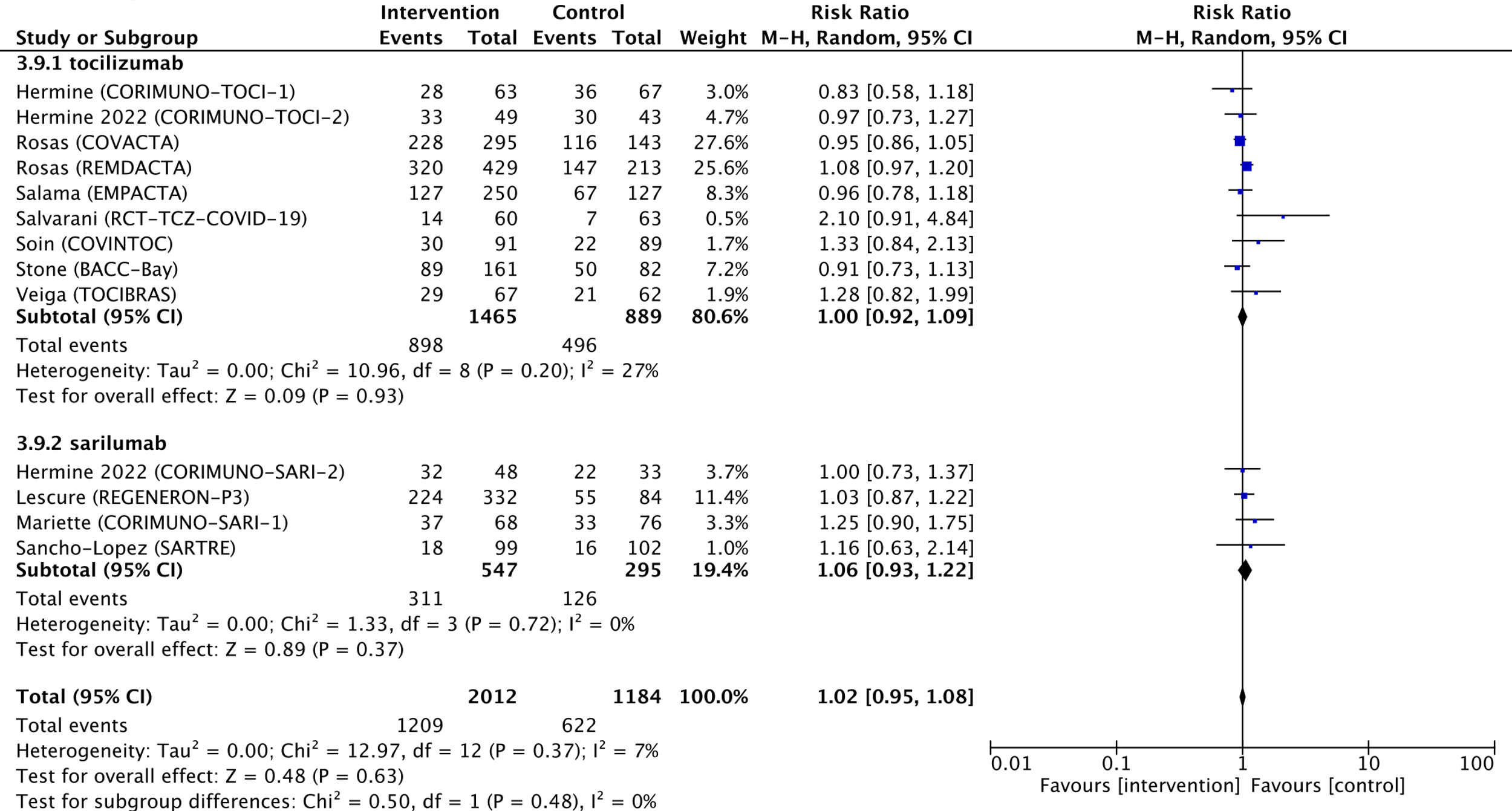


Supplementary Figure 7. time to discharge


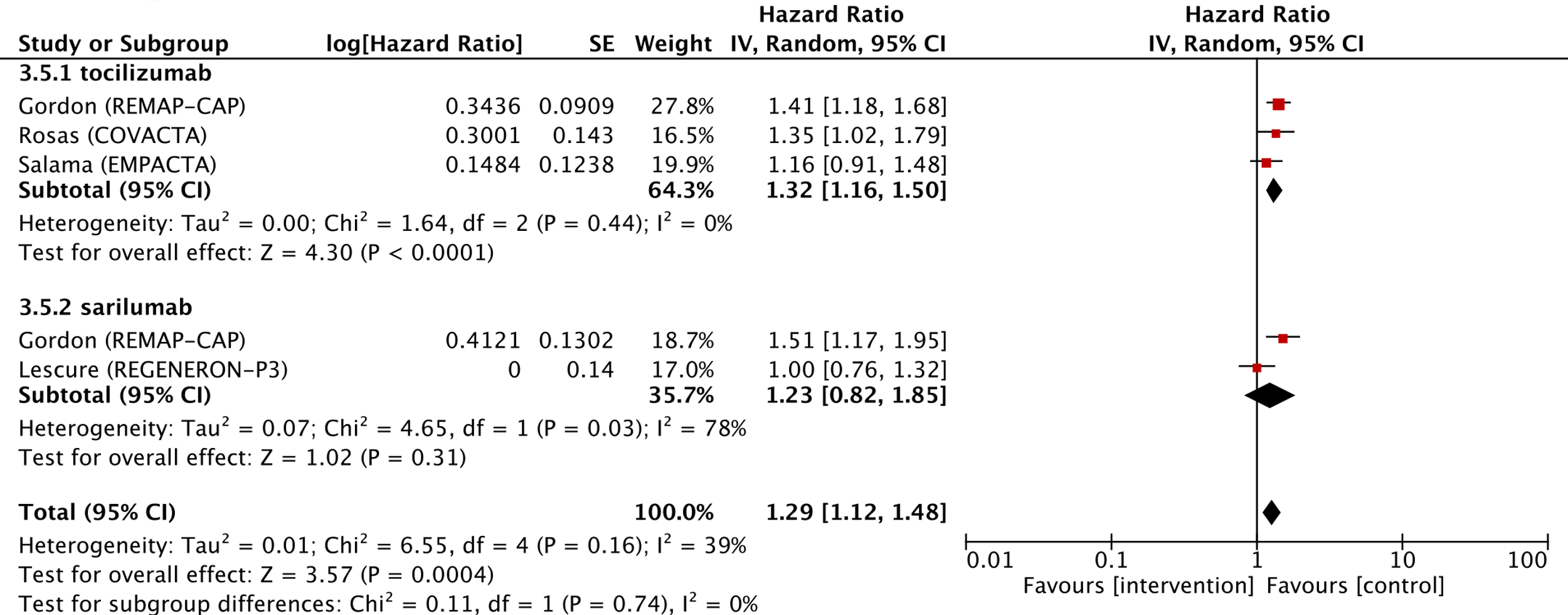


Supplementary Figure 8. hospital discharge


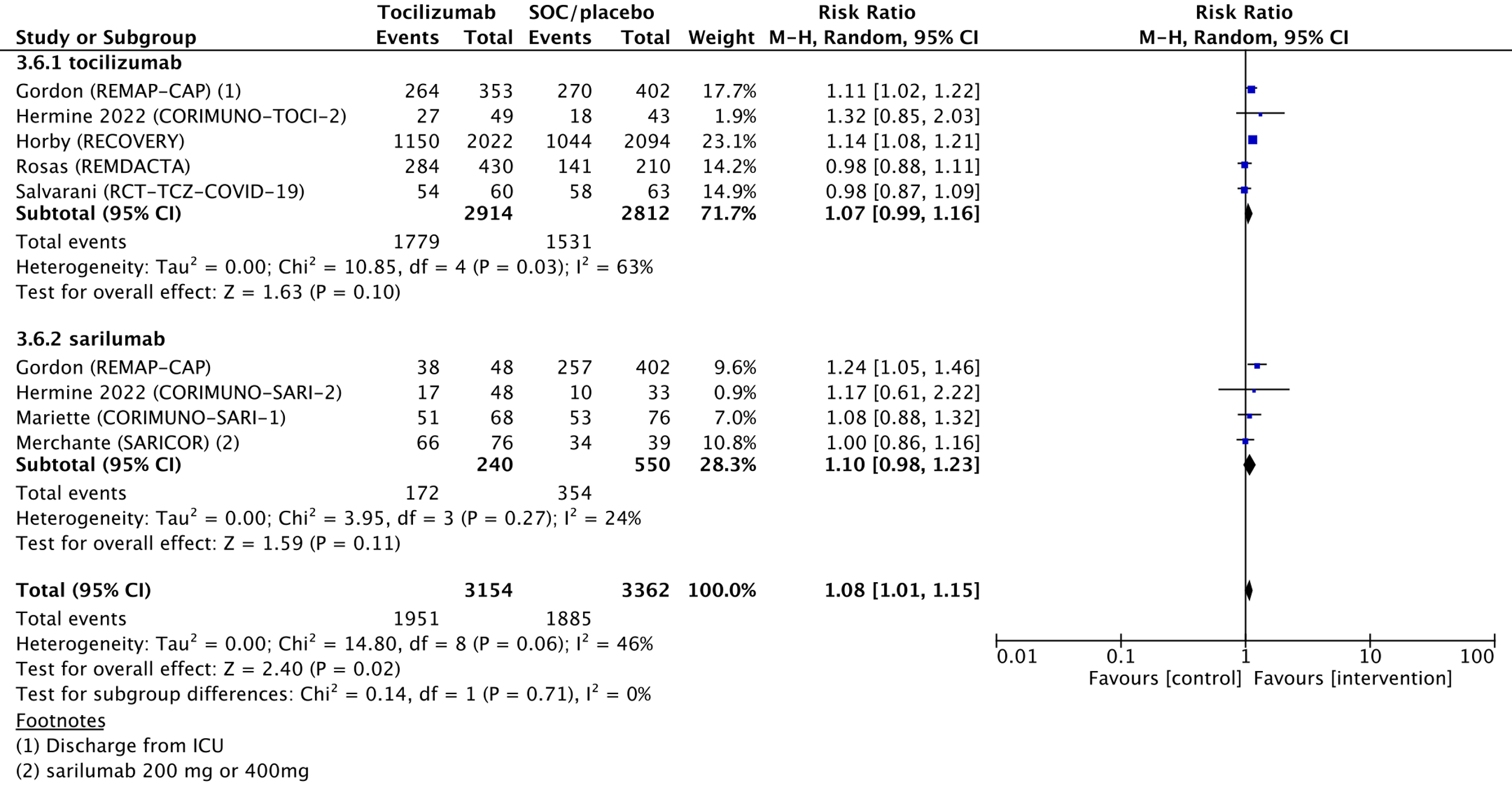


Supplementary Figure 9. intensive care unit admission


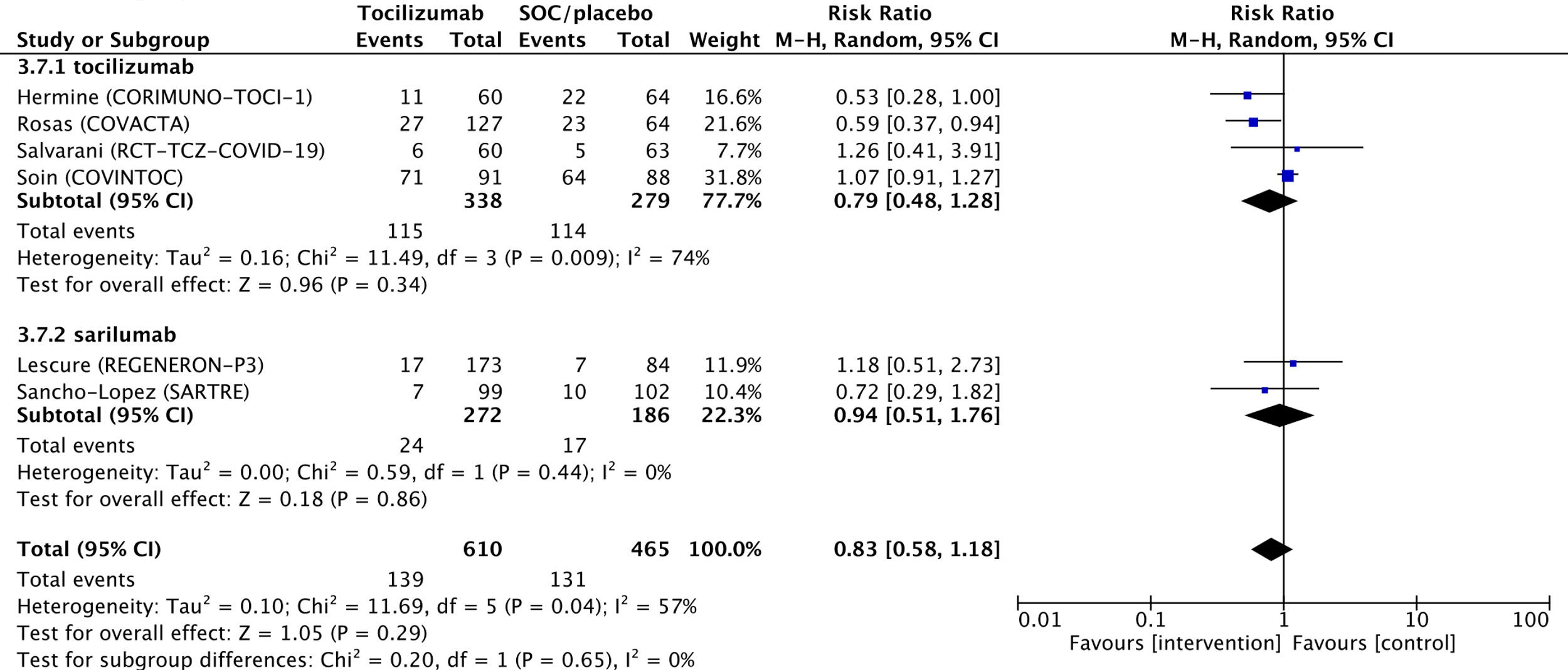


Supplementary Figure 10. time to clinical improvement


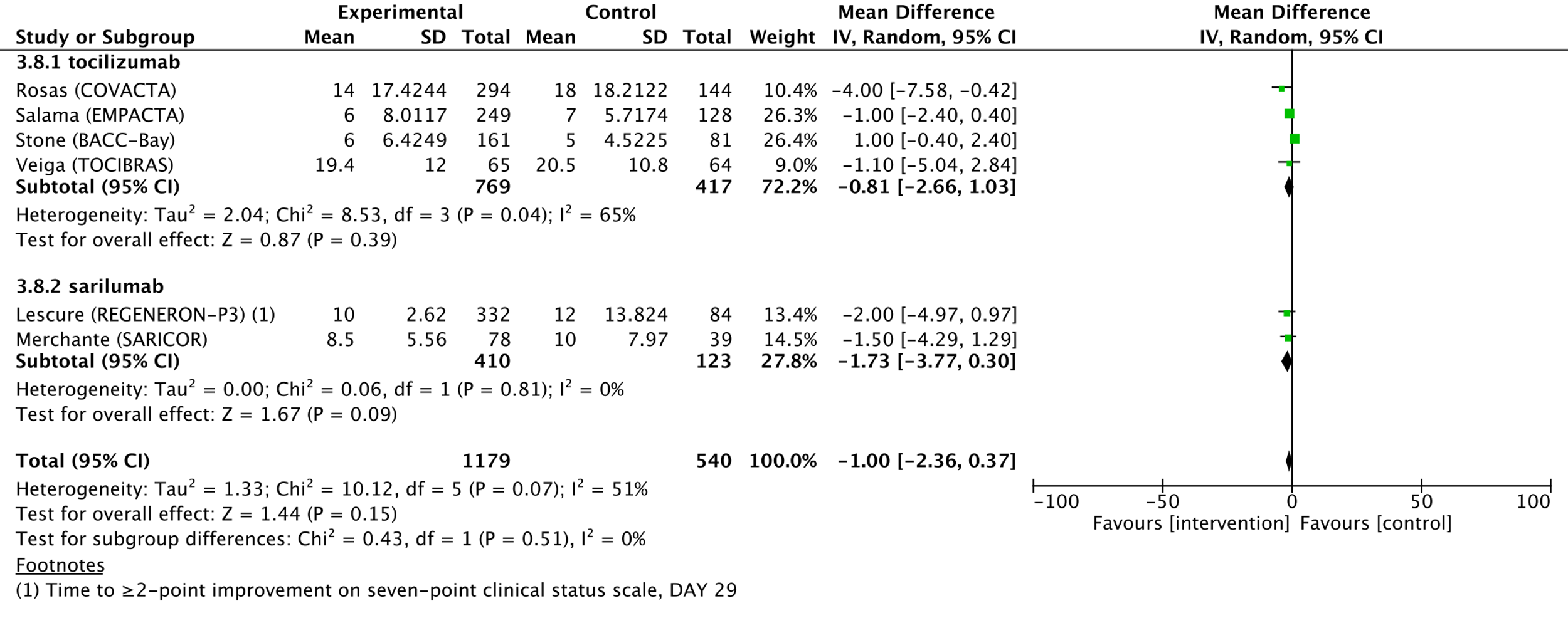


Supplementary Figure 11. 28-day mortality, concomitant steroid therapy cut-off by 50%


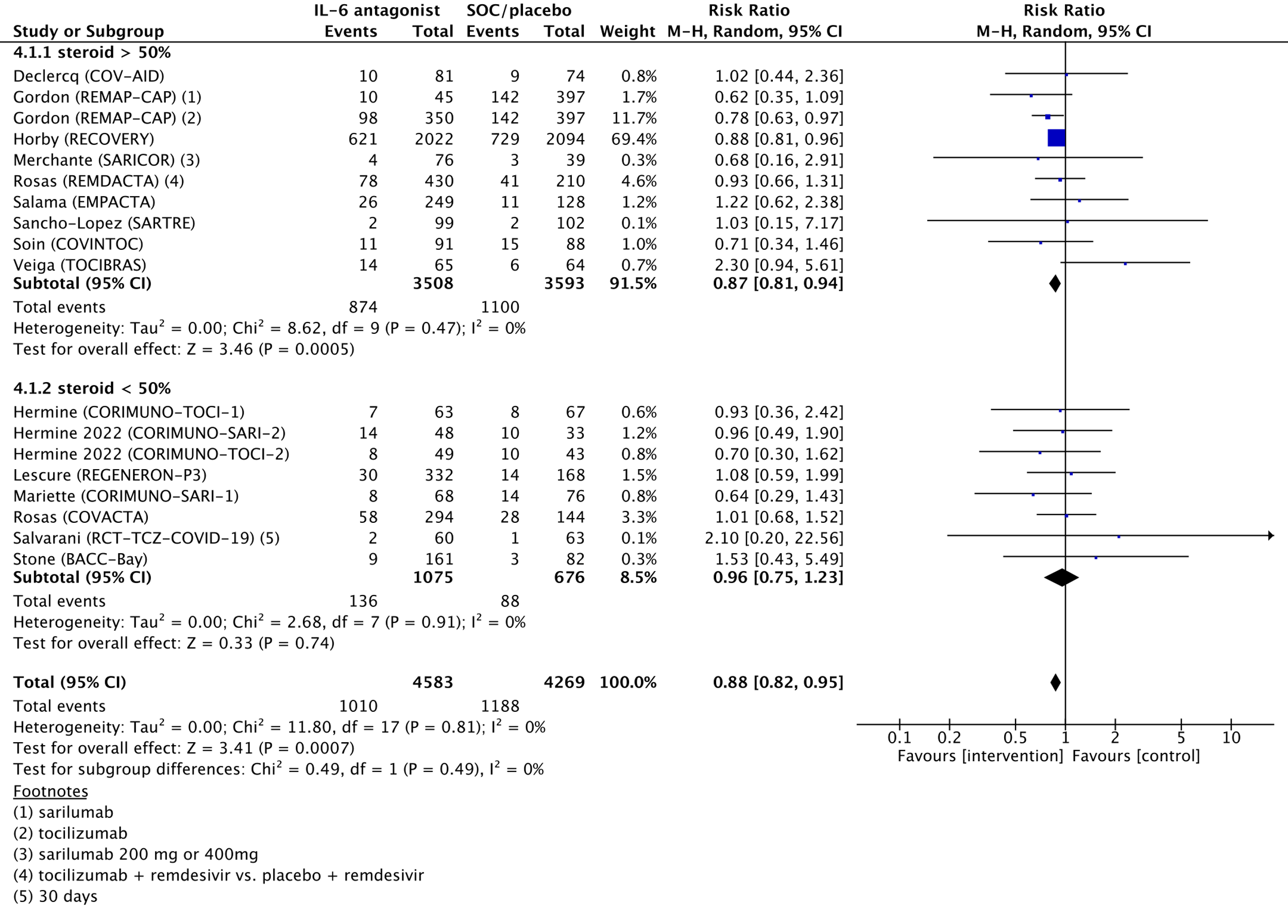


Supplementary Figure 12. progression to IMV, concomitant steroid therapy cut-off by 50%


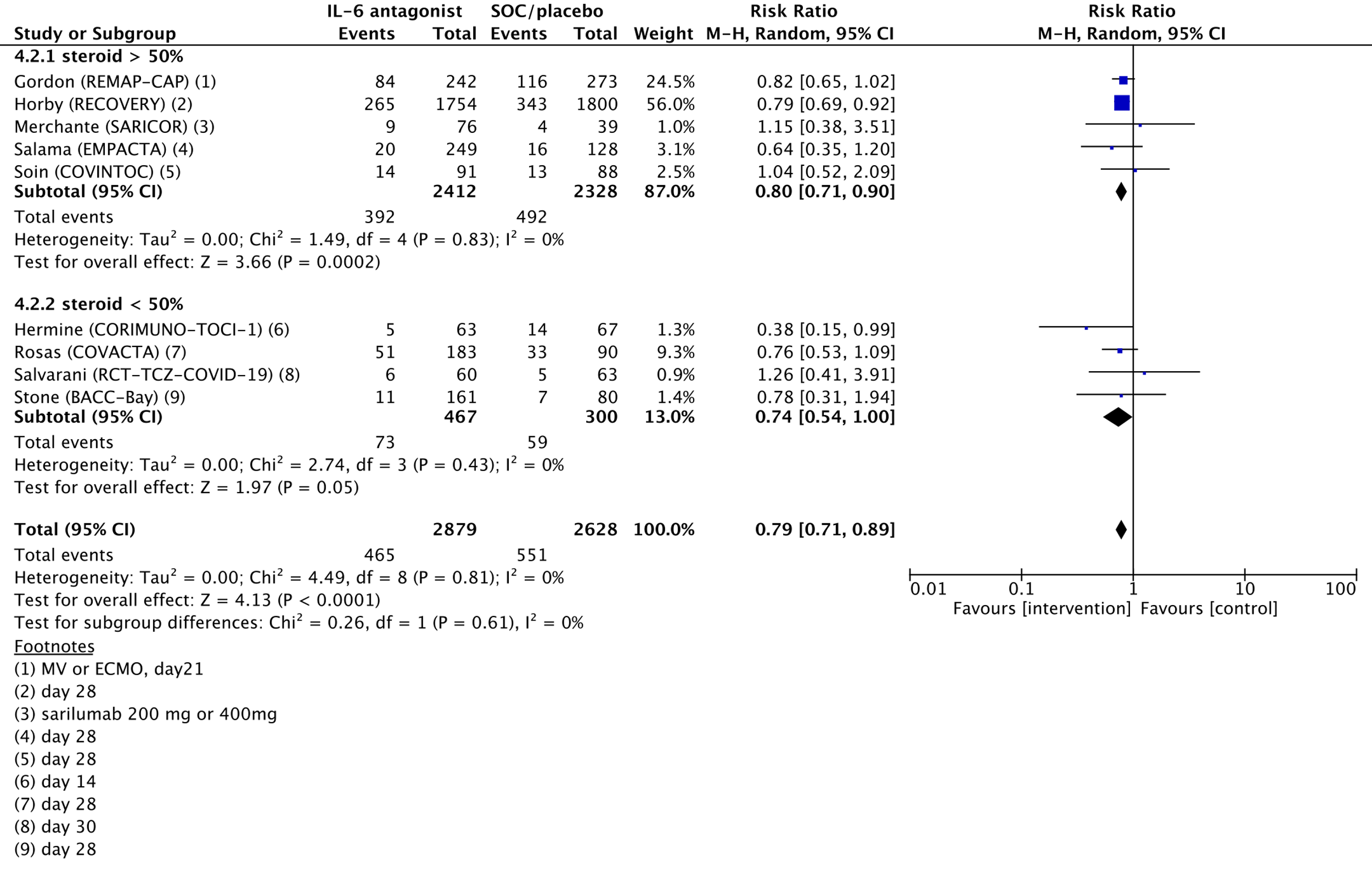


Supplementary Figure 13. serious adverse events, concomitant steroid therapy cut-off by 50%


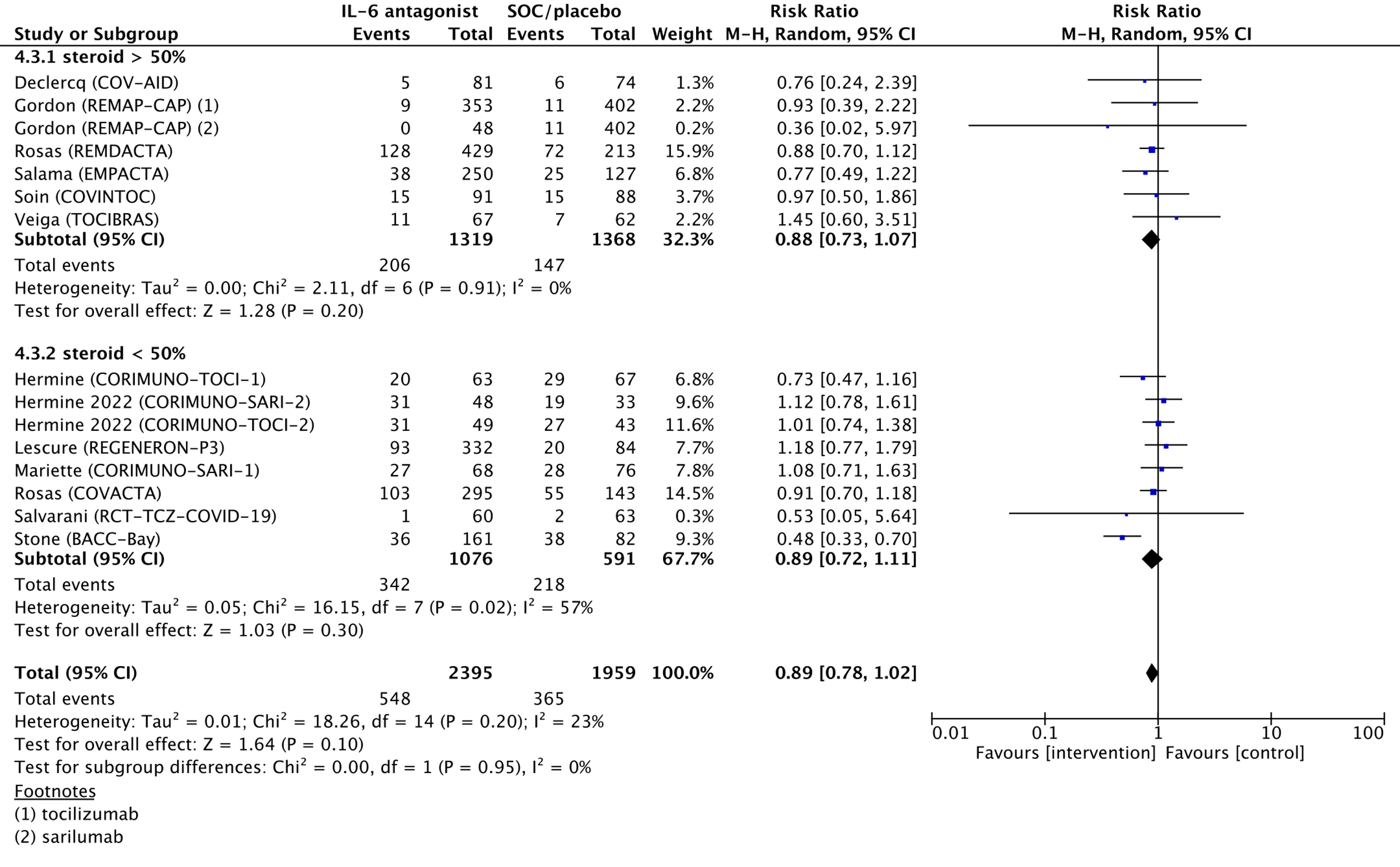


Supplementary Figure 14. secondary infection, concomitant steroid therapy cut-off by 50%


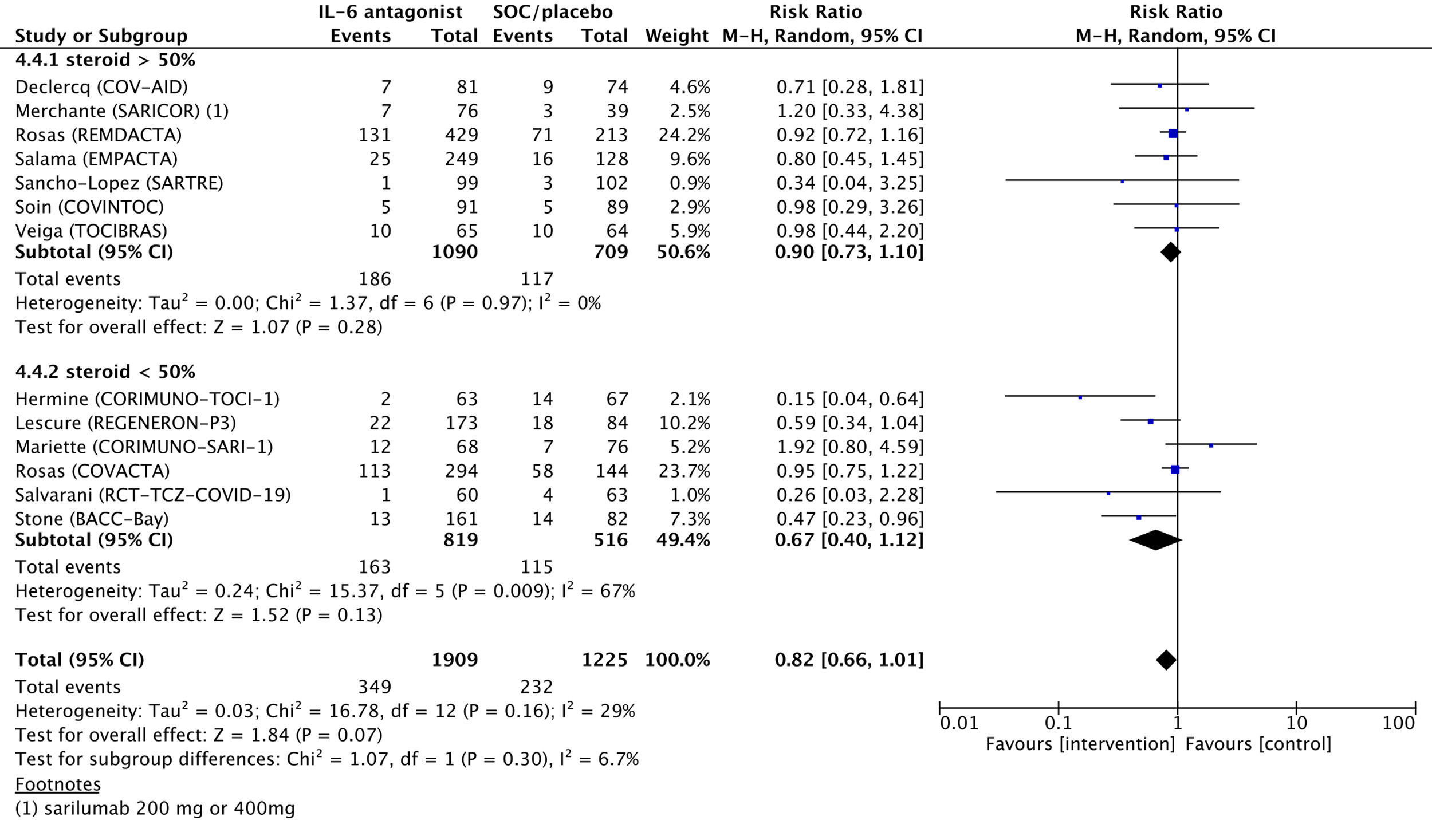

Supplement: Supplemental Material [file TEMI_A_2059405_SM8986.zip › Suppl files/SM_1_Supplement_figures_semifinal_clean copy.docx]
