# Supplementary material for: Clinical efficacy and safety of interleukin-6 receptor antagonists (tocilizumab and sarilumab) in patients with COVID-19: a systematic review and meta-analysis
Source: Emerg Microbes Infect. 2022 Apr 18;11(1):1154–65. doi: 10.1080/22221751.2022.2059405 (PMC9037226; doi:10.1080/22221751.2022.2059405)
Supplement: Supplemental Material [file TEMI_A_2059405_SM8986.zip › Suppl files/SM3+Risk+of+bias+summary.pdf]

	Random sequence generation (selection bias)	Allocation concealment (selection bias)	Blinding of participants and personnel (performance bias)	Blinding of outcome assessment (detection bias)	Incomplete outcome data (attrition bias)	Selective reporting (reporting bias)
Declercq 2021 (COV-AID)	+	+	?	+	+	+
Gordon (REMAP)	+	+	+	+	+	+
Hermine (CORIMUNO-TOCI-1)	+	+	+	+	+	+
Hermine 2022 (CORIMUNO-TOCI-2)	+	+	?	+	+	+
Horby (RECOVERY)	+	+	+	+	+	+
Lescure (REGENERON-P3)	+	+	+	+	+	+
Mariette (CORIMUNO-SARI-1)	+	+	?	+	+	+
Merchante 2022 (SARICOR)	?	+	?	+	?	+
Rosas (COVACTA)	+	+	+	+	+	+
Rosas (REMDACTA)	+	+	+	+	+	+
Salama (EMPACTA)	+	+	+	+	+	+
Salvarani (RCT-TCZ-COVID-19)	+	+	?	?	+	+
Sancho-Lopez (SARTRE)	+	+	+	+	+	+
Soin (COVINTOC)	+	+	?	?	+	+
Stone (BACC-Bay)	+	+	+	+	+	+
Veiga (TOCIBRAS)	+	+	?	?	+	+
