# Supplementary material for: Clinical efficacy and safety of interleukin-6 receptor antagonists (tocilizumab and sarilumab) in patients with COVID-19: a systematic review and meta-analysis
Source: Emerg Microbes Infect. 2022 Apr 18;11(1):1154–65. doi: 10.1080/22221751.2022.2059405 (PMC9037226; doi:10.1080/22221751.2022.2059405)
Supplement: Supplemental Material [file SM+4+Funnel+plot.pdf › Suppl files/SM+4+Funnel+plot.pdf]

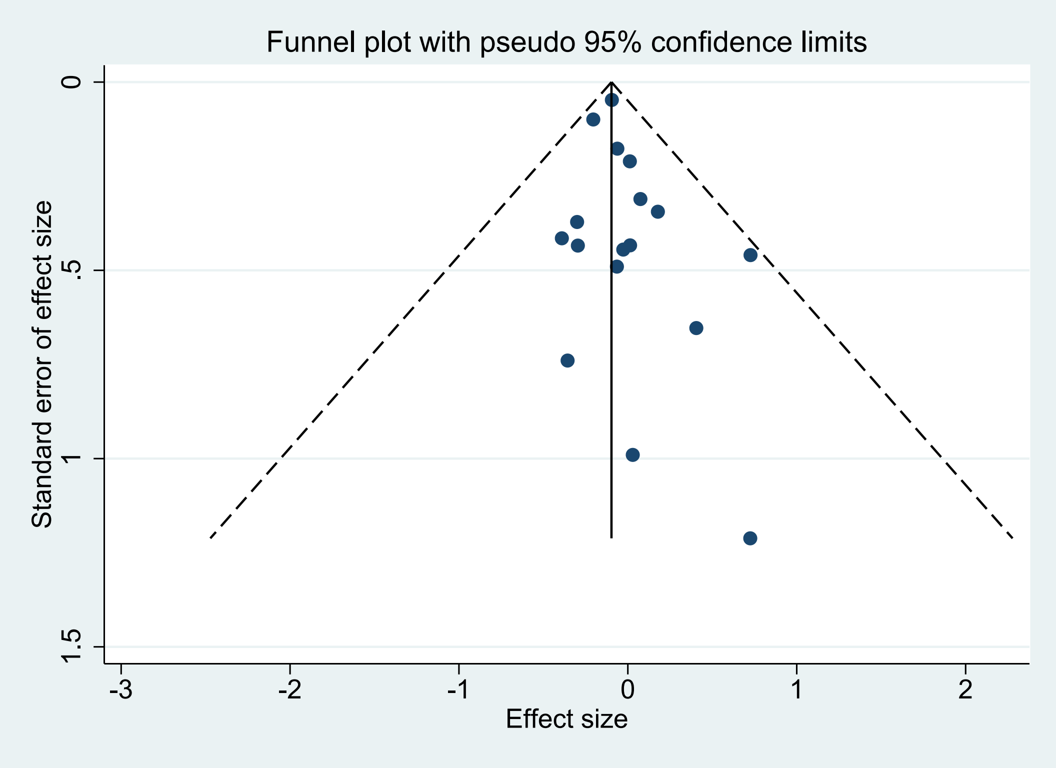
Funnel plot with pseudo 95% confidence limits
Standard error of effect size
0
.5
1
1.5
-3
-2
-1
0
1
2
Effect size
